# Synthesis of cyclic carbonates from CO_2_ cycloaddition to bio-based epoxides and glycerol: an overview of recent development

**DOI:** 10.1039/d3ra03028h

**Published:** 2023-07-26

**Authors:** Muhammad Usman, Abdul Rehman, Faisal Saleem, Aumber Abbas, Valentine C. Eze, Adam Harvey

**Affiliations:** a Department of Chemical and Polymer Engineering, University of Engineering and Technology Lahore, Faisalabad Campus Pakistan a.rehman2@uet.edu.pk; b School of Engineering, Newcastle University Newcastle Upon Tyne NE1 7RU UK; c Songshan Lake Materials Laboratory, University Innovation Park Dongguan 523808 China

## Abstract

Anthropogenic carbon dioxide (CO_2_) emissions contribute significantly to global warming and deplete fossil carbon resources, prompting a shift to bio-based raw materials. The two main technologies for reducing CO_2_ emissions are capturing and either storing or utilizing it. However, while capture and storage have high reduction potential, they lack economic feasibility. Conversely, by utilizing the CO_2_ captured from streams and air to produce valuable products, it can become an asset and curb greenhouse gas effects. CO_2_ is a challenging C1-building block due to its high kinetic inertness and thermodynamic stability, requiring high temperature and pressure conditions and a reactive catalytic system. Nonetheless, cyclic carbonate production by reacting epoxides and CO_2_ is a promising green and sustainable chemistry reaction, with enormous potential applications as an electrolyte in lithium-ion batteries, a green solvent, and a monomer in polycarbonate production. This review focuses on the most recent developments in the synthesis of cyclic carbonates from glycerol and bio-based epoxides, as well as efficient methods for chemically transforming CO_2_ using flow chemistry and novel reactor designs.

## Abstract 2

Anthropogenic carbon dioxide (CO_2_) emissions contribute significantly to global warming and deplete fossil carbon resources, prompting a shift to bio-based raw materials.

## Graphical abstract

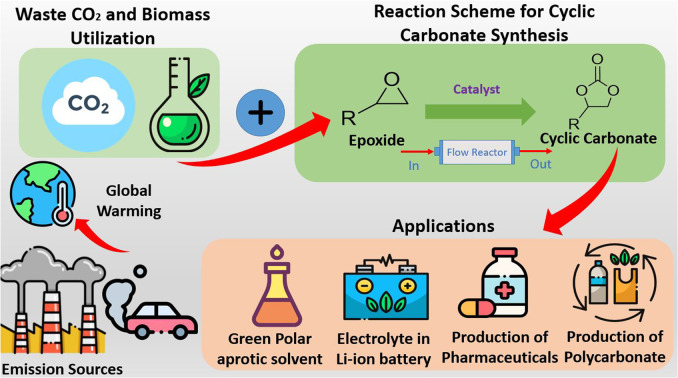

## 1. Introduction

**Fig. 1:**
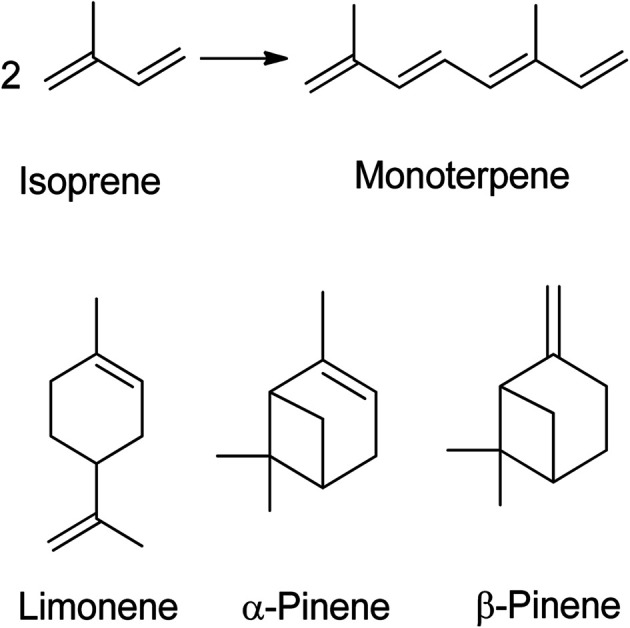
Two isoprene units give monoterpene, the chemical structure of limonene and pinene.^1^

A terpene is a hydrocarbon consisting of at least two isoprene units (C_5_H_8_) and is odourless. Five carbon atoms with double bonds make up an isoprene molecule, also referred to as an isoprene unit. Isoprene itself, a gaseous hydrocarbon, is emitted by leaves as a natural by-product of plant metabolism. Monoterpenes (C_10_H_16_) contain two isoprene units (Fig. 1), while sesquiterpenes have three, diterpenes have four, triterpenes have six, and tetraterpenes have eight isoprene units.^1^ The name of a terpene always ends with –ene. The structure of terpenes determines whether they are cyclic or acyclic, with cyclic terpenes forming a ring and acyclic terpenes being linear. Most terpenes are monoterpenes, which can be categorized into groups based on their structure. Terpenes have low molecular weight, which enables them to evaporate quickly. Terpenes are sometimes confused with terpenoids, but the key difference is that terpenes do not contain oxygen while terpenoids do.

Monoterpenes are volatile and commonly used for flavouring and perfumes due to their strong odour. They are also lipophilic, allowing them to easily penetrate cell membranes, and are known to have antispasmodic properties.^2–4^ Cannabis contains approximately 200 terpenes, which is only a small fraction of the 20 000 terpenes known to exist in plants.^5^ Among these, limonene and pinene are major terpenes.^6^ Limonene, a colourless liquid, has a boiling point of 175 °C and is comprised of two isomers: d-limonene and l-limonene. d-limonene is the primary component of essential oils found in citrus fruit rinds, such as oranges, lemons, mandarins, grapefruits, and limes. On the other hand, l-limonene is emitted as a key component of volatile oils by oaks and pines.^7^ Orange peels contain around 97% of limonene,^8^ and with around 110 million tons of citrus fruits produced annually, this waste can be a source of essential oil extraction. Various methods, such as solvent extraction, steam distillation, and water distillation extraction, have been employed to extract the oil from orange peels. Steam distillation has been found to produce the maximum yield of oil at 4.40%, followed by water distillation extraction at 3.47% and solvent extraction at 2.53%.^9^

d-Limonene is widely manufactured globally, with approximately 300 orange oil distillation plants operating worldwide to produce technical-grade (95%) and food-grade (96%) d-limonene from orange oil.^7^ The market for d-limonene was estimated to be worth USD 473.72 million in 2021, and it is anticipated to increase to USD 694.59 million between 2022 and 2029, increasing at a compound annual growth rate (CAGR) of 4.90%.^10^ Limonene is mainly used in the flavour and fragrance industry, as a solvent, and for making polymers and adhesives.^11^ It was first used in 2008 to obtain fats and oils, replacing toxic solvents like *n*-hexane, effectively eliminating toxicity and environmental impact. d-Limonene is also an excellent cleansing agent, with a high KB (74) and density of 0.84, which allows it to hold a large amount of dirt before becoming ineffective. A compound's KB value is a measure of its ability to hold dirt.^7^ Additionally, l-limonene and d-limonene are among the 44 sustainable solvents developed by GlaxoSmithKline (GSK).^12^

In Kraft wood pulping, turpentine oil is produced as an energy recovery oil. The increasing demand for chemical products containing artificial flavours, fragrances, and solvents is driving the demand for turpentine oil, which is also a promising alternative fuel for various industrial applications.^13^ Turpentine oil is currently the primary source of pinene, with an average yield of 0.3–1 kg per tonne of pulp production. Worldwide, the pulping industry produces an estimated 3.5 × 10^5^ Mt per year of turpentine oil, with α-pinene accounting for 70% of the total monoterpene content.^14,15^ α-Pinene is the dominant form of pinene, found in pine trees and cannabis, and responsible for the characteristic pine scent. The majority of applications use α-pinene. Pine-sol, a cleaning product, contains this terpene. α-Pinene has significant potential as an engine biofuel, as it is comparable to rocket fuel in terms of volumetric heating.^16^

Glycerol is a versatile chemical that may be processed through numerous paths to creating a variety of products with value-added, such as reforming, dehydration, esterification, selective oxidation, hydrogenolysis, and acetylation.^17^ However, a direct reaction of glycerol and CO_2_ is an attractive route that effectively combines two waste products to produce glycerol carbonate. The production of glycerol is a by-product of the biodiesel or green diesel industry, where approximately 10 wt% of glycerol is obtained from every kg of biodiesel, and around 70% of glycerol is produced in this manner.^18^ The biodiesel market was estimated to be worth USD 40.6 billion in 2021 and is anticipated to increase by 4.4% to USD 52.7 billion by 2027.^19^ With the expected increase in biodiesel production, there will be a surplus of glycerol that can be utilized as a low-cost chemical feedstock to produce glycerol carbonates and glycerol acetates (also known as acetins). Acetins are non-natural, synthetic products that can be obtained either through traditional chemical conversion or new biological conversion. Chemical conversions involve toxic solvents and reactants (such as acetic acid and acetic anhydride) and require harsh temperature and pressure conditions, making them non-green and polluting. In contrast, biological processes for acetin production are considered green as they use enzymes or microbes as biocatalysts. Acetins have various applications, such as plasticizers, explosives, emulsifiers, food additives, and fuel additives.

Both terpenes and glycerol offer pathways for the conversion of low-value compounds into high-value chemicals with diverse applications. Terpenes, such as limonene, can be coupled with CO_2_ to produce terpene-based cyclic carbonates, while glycerol can be transformed into glycerol carbonate (GC). Glycerol carbonate (GC) is a liquid with a high water solubility, high boiling point, and low toxicity. It has potential applications as a solvent, as a surfactant, and as an electrolyte in Li-ion batteries.^20,21^

### 1.1 CO_2_ and its utilisation

In 2020, global CO_2_ emissions reached 34.8 Gt, resulting in a reported temperature increase of 1.16 °C relative to 1920.^22^ In April 2022, global monthly mean CO_2_ emissions were at a record high of 418.39 ppm, up from 415.82 ppm in April 2021.^23^ It is projected that by 2050, 5.6 Gt per year of CO_2_ will need to be captured and stored, equivalent to 40 Mt year of CO_2_ today.^24^ However, currently, only 100 Kt of cyclic carbonates are produced annually, consuming 40 Kt of direct CO_2_ each year.^25,26^ Approximately 35 Mt of CO_2_ is released each year, with less than half being emitted by large point sources that can be captured, stored, and utilized.^27^ To address this global CO_2_ challenge, a multifaceted approach is necessary, which includes Carbon Capture and Storage (CCS) primarily focusing on capturing and storing, Carbon Capture and Utilization (CCU) focusing on utilization after capturing, energy transition to non-fossil fuels such as solar, wind, and tidal, aiming to improve energy efficiency and minimizing heat loss.^25^

There are now competitive technologies such as solar and wind that can replace fossil fuels.^28^ However, renewable energy resources are intermittent, posing a significant concern.^29^ To reduce greenhouse gas (GHG) emissions, efforts are being made to replace fossil fuel-based power plants and synthesis gas generators with biomass-based processes. Nevertheless, bio-based fuel requires being used as a combined mixed fuel due to its low energy density. Thus, conventional power generation may need to be employed to meet peak demands. Moreover, most chemical and fuel production currently rely on fossil fuels as feedstocks, necessitating technologies to reduce negative emissions associated with these processes.^30^

### 1.2 Methods of CO_2_ utilisation

Two main technologies that have been deployed for CO_2_ emission reduction are CCS and CCU. CCS captures and stores CO_2_ in large quantities, while CCU methods valorize waste and renewable CO_2_ as inputs for added-value products.^31^ Both technologies involve CO_2_ capture, compression, and storage or utilization using catalytic technologies. However, CCS consumes a lot of energy and provides a negative return on investment due to the associated cost of capturing and storing, whereas CCU consumes less energy and provides a net positive ROI because the product sold after manufacturing gives a profit.^32^ Climate change mitigation is the primary objective of CCS, whereas resource security is the main objective of CCU. The focus of current research is on developing CO_2_ utilization methods that consume less energy.

CCS targets large source points or ambient air to capture a large amount of CO_2_, while chemical production quantities vary regionally in CCU, limiting the CO_2_ demand. CCS deployment currently has few economic incentives, while incentives are already provided for CCU to a variety of actors. By minimizing the cost of CO_2_ capture, CO_2_ can become an appreciating asset rather than a liability.^33^ However, it's important to note that CO_2_ stored in CCS is intended to last more than a thousand years, while CCU stores CO_2_ in the product only temporarily, which is released at the end of its life cycle back into the atmosphere. CSS is more mature and well-established, while CCU is less familiar and in the early development stages, but the latter is regarded as more positive than the former due to the associated risk of storage and transport with the former technology.^34^

CSS, which captures and compresses CO_2_, costs 80% of the total cost associated with the process. Research and development efforts must aim to reduce these costs. Currently, technologies such as pre-combustion, post-combustion, and oxy-fuelling can capture CO_2_ from cement, steel, iron, and biogas sweetening processes.^35^ Once captured and separated, CO_2_ should be transported cost-effectively through pipelines to the injection station. To reduce corrosion and surplus costs, impurities and moisture must be removed. CO_2_ must be compressed into supercritical form and monitored after injection to prevent it from seeping out and remaining confined.^36,37^ Long-term sequestration is preferred in active or depleted gas reservoirs and deep aquifers.^38^ According to the Intergovernmental Panel on Climate Change (IPCC) and Special Report on Emission Scenarios (SRES), CCS may reduce global CO_2_ emissions by 17% by 2050 and is one of the least expensive strategies for doing so in the near future.^39,40^

CO_2_ is a challenging C1-building block because of its high kinetic inertness and thermodynamic stability, with a standard heat of formation of Δ*H*_f_ = −394 kJ mol^−1^ and a standard Gibbs energy of Δ*G*_f_ = −395 kJ mol^−1^.^41^ To react effectively to epoxide and CO_2_, a catalyst is needed. Metal-containing catalysts can create cyclic carbonate and polycarbonate depending on the nucleophile species, metal species, nucleophile-to-metal ratio, and reaction conditions, but metal-free catalysts are often more selective towards cyclic carbonates. Optimization is necessary to maximize production. Two broad categories of CO_2_ utilization processes exist physical and chemical. To be physically utilised, CO_2_ molecules must maintain their identity, either as a distinct entity or suspended in a solution. These processes, which include carbonated drinks, fire extinguishers, dry ice, enhanced oil recovery (EOR), enhanced gas recovery (EGR), and enhanced geothermal systems, are based on the physical properties of CO_2_. Some physical utilization processes, such as EOR and EGR, have the potential to store large volumes of CO_2_ and are called sequestration processes.^42^ Chemical utilization involves CO_2_ being converted into a new compound. The chemical potential of CO_2_ can be utilized to produce fuels and chemicals. This article focuses on chemical utilization because of its limitless potential to abate CO_2_. Several prominent chemical utilization pathways, such as those summarized in Table 1, are available to convert CO_2_ chemically into useful products, with varying production rates, technology readiness levels (TRL), the specific mass (CO_2_ utilization in ton per ton of product), and CO_2_ uptake potential.^43–47^

**Table 1:** Summary of chemical CO_2_ utilization options based on product

Product	Current production (Mt per year)	Technology readiness level (TRL)	Specific mass (CO_2_ utilization in ton perton of product)	CO_2_ uptake (MtCO_2_ per year)	Reference
Urea	180	9	0.74	132.3	43
Salicylic acid	0.17	9	0.32	0.054	44
Methanol	65	9	1.37	89.24	44
Polycarbonate	5	9	0.17	0.87	43
Polyurethane	15	9	0.30	4.5	45
Ethylene carbonate	0.2	8	0.50	0.099	45
Algae	35	7	1.8	63	46
Calcium carbonate	113.9	7	0.44	113.9	45
Methane	1100–1500	7	2.75	3050–4150	44
Propylene carbonate	0.2	7	0.43	0.086	45
Syngas	359	6	1.47	526.55	47
Sodium carbonate	62	6	0.42	25.73	44
Dimethyl carbonate	1.60	5	1.47	2.35	45
Magnesium carbonate	20.50	4	0.26	5.35	44
Acetic acid	10.25	3	0.73	7.51	44
Acrylic acid	5.85	3	0.61	3.57	44
Formaldehyde	21	3	1.45	30.45	45
Dimethyl ether	11.4	3	1.91	21.79	45
Ethanol	80	2	1.91	152.88	44

Technologies for producing urea, salicylic acid, methane, methanol, ethylene carbonate, polyurethanes, and polycarbonates have a technology readiness level (TRL) of 9, indicating that they are well-established and mature. Other technologies listed are currently maturing and developing, while some are still immature and in the early stages of development. The CO_2_ uptake potentials mentioned do not include emissions associated with processing. However, the reduction that can be achieved if environmentally benign processes are developed and deployed for utilization is an overview of the overall reduction. Currently, approximately 220 Mt per year of CO_2_ is utilized for producing different chemicals, excluding methane, syngas, calcium carbonate, sodium carbonate, ethanol, *etc.* Urea production consumes the majority of CO_2_, followed by salicylic acid, cyclic carbonate, and polycarbonate. The remaining CO_2_ is used for methanol, ethanol, *etc.* Additionally, around 20 Mt per year of its usage as a technical fluid must be added to this amount. It is estimated that today, the chemical industry consumes 92% of the approximately 240 Mt per year of CO_2_ that is consumed.^48^

Over the next decade, a growing synthetic technology industry may increase the rate at which CO_2_ is utilized to 350 Mt per year. By 2040, we could see a surge in the consumption of CO_2_ for chemicals, rising to around 1000 Mt per year, potentially making a considerable difference.^49^

### 1.3 Industrial processes of CO_2_ utilizations

**Scheme 1:**
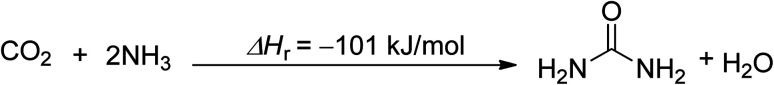
Synthesis of urea (reproduced from ref. 26 with permission from Wiley-VCH copyright 2021).

The first mature method for CO_2_ chemical utilization is urea production, which consumes 0.735 to 0.75 tons of CO_2_ per ton of urea and is highly exothermic (Scheme 1).^28,37^ The global urea market has reached 180 Mt per year in 2021, with a predicted growth of 0.8% between 2022 and 2027.^50^ A novel process has been proposed to produce urea from power plants and electricity simultaneously by using CO_2_ in the flue gas as a reactant for urea production, with 1 ton of CO_2_ utilization producing 1.68 tons of urea.^51^

**Scheme 2:**
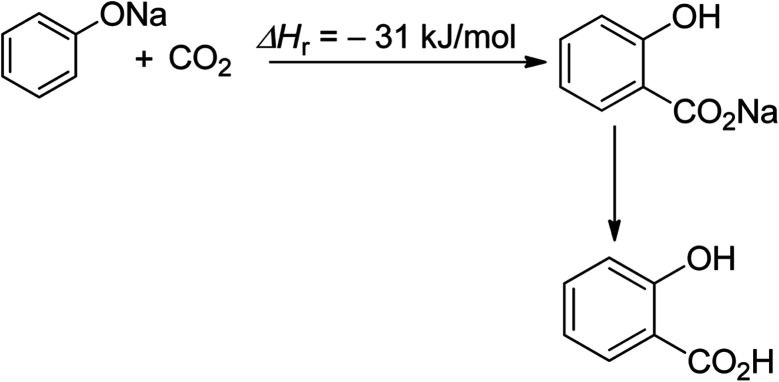
Synthesis of salicylic acid (reproduced from ref. 26 with permission from Wiley-VCH copyright 2021).

Salicylic acid synthesis is a second example of efficient CO_2_ utilisation (Scheme 2).^52^ The commercialization of this process dates back to the 19th century, and salicylic acid production currently consumes 29 Kt of CO_2_ directly, with a total production of 90 Kt per year.^27,53^

**Scheme 3:**
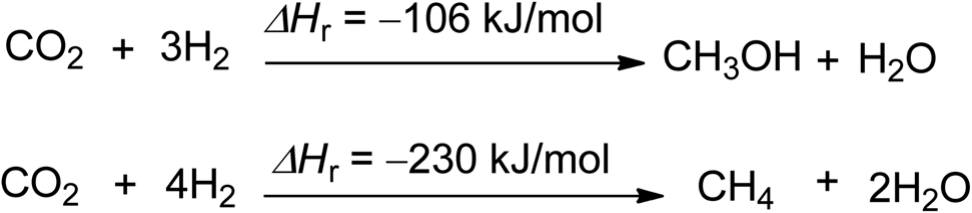
Synthesis of methanol and methane using CO_2_ and hydrogen (reproduced from ref. 26 with permission from Wiley-VCH copyright 2021).

The production of carbon-neutral fuels like methanol and methane is the third efficient chemical use of CO_2_ (Scheme 3).^54,55^ These fuels have zero CO_2_ emissions when combusted under certain conditions, and their potential scale is almost limitless, as they can replace liquid and gaseous fossil fuels. Methane and methanol production has the potential to reduce 2.75 and 1.373 tons of CO_2_ per ton of product produced, respectively.^44^ Hydrogen is the main reactant needed for the feasibility of methanol and methane production, and methane can also be used for the production of carbon black and rubber.^56^

**Scheme 4:**
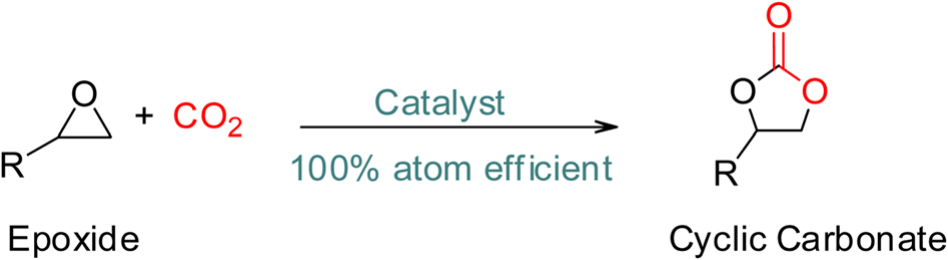
Synthesis of cyclic carbonates from epoxide and CO_2_.^32^

The production of polycarbonates is the fourth illustration of efficient CO_2_ utilisation, which uses renewable, economical, and non-toxic CO_2_ instead of the poisonous phosgene route (Scheme 4).^57,58^ Similarly, the fifth example of effective CO_2_ utilization is the production of polyurethanes (PU),^59^ which have dominated coatings, adhesives, sealants, and elastomers for the past three to four decades and can be tailored for different applications (Scheme 4).^60–62^ The current production rate of polycarbonates and polyurethanes is 5 and 15 Mt per year, respectively.^45,47^

**Fig. 2:**
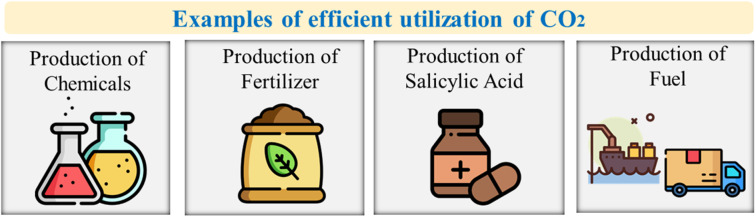
Examples of effective utilization of CO_2_ and applications of cyclic carbonates.^26^

Other developing CO_2_ utilization technologies include dimethyl carbonate (DMC), dimethyl ether (DME), ethanol and algae. Ethanol is produced conventionally by fermenting sugars or corn, and it has the potential to be used as a zero-emission fuel, but the process design and low catalyst activity remain challenges.^61,62^ DME is an alternative green fuel that results in fewer GHGs and can coproduce electricity, while DMC has potential as a methylating agent but faces catalyst selection issues (Table 1).^63,64^ Algae cultivation has great potential as a biomass source for green fuel production and can absorb CO_2_ and convert it to oxygen for water purification.^65^Fig. 2 shows examples of mature CO_2_ utilization technologies and applications of cyclic carbonates.

## 2. Cyclic carbonates

The production of five-membered cyclic carbonates through the reaction of epoxides and CO_2_ is a commercially viable green and sustainable chemistry reaction (Scheme 4).^32^ This process uses CO_2_ as a readily available, non-toxic, renewable reactant, which is one of its key advantages. Additionally, it is a 100% atom-efficient reaction as the product is comprised of all reactants, with no need for a solvent. The reaction is also thermodynamically favourable, as epoxides have greater free energy than CO_2_, which offsets the required thermodynamic stability of CO_2_.^66^ Therefore, cyclic carbonates production from CO_2_ and epoxides is a promising and environmentally friendly approach to chemical synthesis.

**Fig. 3:**
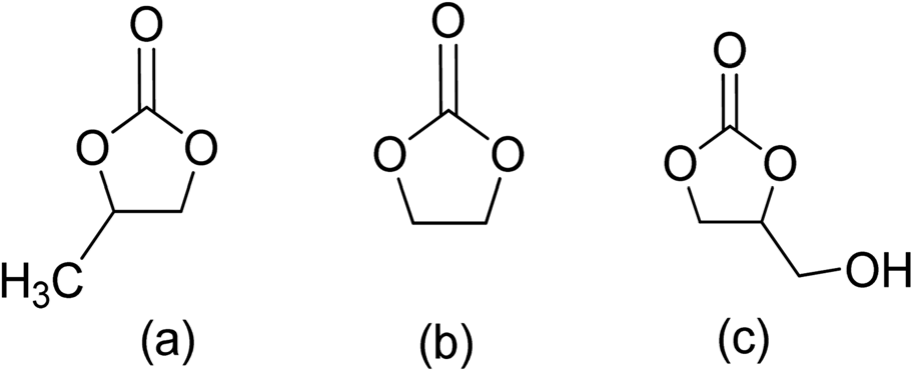
Cyclic carbonates: (a) propylene carbonate (PC), (b) ethylene carbonate (EC), (c) glycerol carbonate (GC).^66^

Cyclic carbonates are considered green solvents, with propylene carbonate (PC), ethylene carbonate (EC), and glycerol carbonate (GC) being the most widely used (Fig. 3.).^66^ The physiochemical properties of cyclic carbonates are listed in Table 2.^66–73^ EC and PC are the most commonly used globally.^74,75^

**Table 2:** Physio-chemical properties of cyclic carbonates

Cyclic carbonates	Propylene carbonate (PC)	Ethylene carbonate (EC)	Glycerol carbonate (GC)
Formula	C_4_H_6_O_3_	C_4_H_6_O_3_	C_4_H_6_O_3_
CAS number	108-32-7	96-49-1	931-40-8
IUPAC name	4-Methyl-1,3-dioxolan-2-one	1,3-Dioxolan-2-one	4-Hydroxymethyl-1,3-dioxolan-2-one
Molecular mass, [g mol^−1^]	102	88	118
Specific gravity [g cm^−3^]	1.20 (20 °C)	1.32 (40 °C)	1.40 (25 °C)
Boiling point, *T*_b_ (°C)	242	248	354
Freezing point, *T*_f_ (°C)	−49	36	−69
Flash point (°C)	135	145	>204
Vapour pressure [kPa]	0.131 (50 °C)	0.003 (25 °C)	n.a
Viscosity, (cP or mPa s at 25 °C)	2.53	1.93	85.40
Solubility (at 20 °C) (mg kg^−1^)	Very soluble	Soluble	Miscible
Hazards	Induces severe eye irritation	A prolonged or repeated exposure can cause serious eye irritation, and cause injury to the kidneys if swallowed	Ingestion or skin absorption can be harmful

### 2.1 Applications of cyclic carbonates

Propylene carbonate (PC) is a highly desirable polar aprotic solvent due to its high dipole moment, high dielectric constant, odorlessness, colourlessness, and low viscosity.^68,76^ Additionally, it remains liquid across a wide temperature range and is biodegradable, hydrolyzing in air and water. PC is also non-toxic, non-flammable, and corrosion-resistant, making it an excellent solvent from a health and safety standpoint.^66,68,77^ PC ranks highly on the GSK solvent suitability ranking, making it a green solvent. However, it has a high boiling point, which requires energy-intensive distillation for separation, and therefore, it may not be considered a green solvent under all circumstances.^78^ Despite this, PC can replace harmful polar aprotic solvents affordably and sustainably. EC is not used as a solvent due to its solid nature, so it is combined with other compounds to overcome this issue. Several other cyclic carbonates, such as 1,2-limonene carbonate (1,2-LC) and styrene carbonate, have the same restraint.^66^ Glycerol carbonate (GC) has similar properties and applications, but its use as a solvent is limited by its high viscosity.^77^

**Fig. 4:**
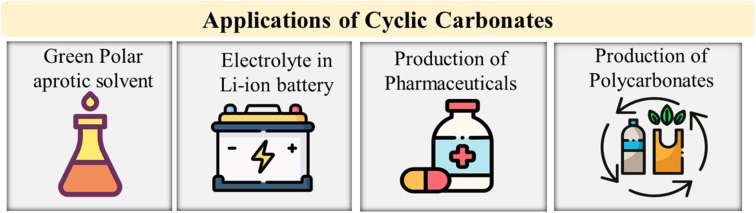
Applications of cyclic carbonates.^32^

Due to their dielectric characteristics, cyclic carbonates make excellent electrolytes for Li-ion batteries.^79^ Li-ion batteries have become popular due to their use in portable devices.^80^ Among Li-based battery electrolytes, EC and PC are commonly used as solvents. Lithium salts can dissolve in cyclic carbonate, providing the high dielectric constant needed for conductive electrolytes.^25^ PC and EC have various uses such as cleaning, paint stripping, degreasing, and the production of paints and coatings, lubricants, dyes, cosmetics, and personal care products.^74,75^ Cyclic carbonates can also be employed as monomers in the production of polymers like polycarbonate and polyurethanes without isocyanate.^59,81^ Cyclic carbonates are more effective than urea or salicylic acid at sequestering CO_2_ in electronic devices, which typically have a lifespan measured in years. Electric cars may have a lifespan of decades, making cyclic carbonates even more effective at sequestering CO_2_.^26^Fig. 4 represents the applications of cyclic carbonates.

### 2.2 Terpene-based cyclic carbonates

**Fig. 5:**
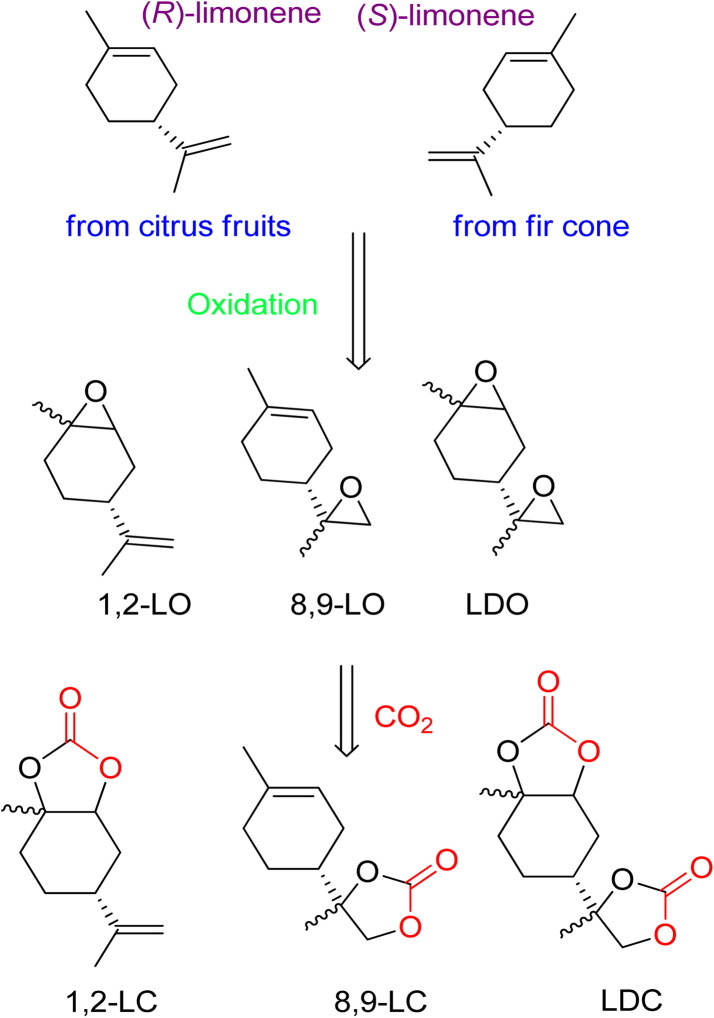
Structure of both (*R*)-(+)-limonene enantiomers, oxidation to afford limonene-based oxides and reaction with CO_2_ to obtain corresponding cyclic carbonates, reproduced from (reproduced from ref. 82 with permission from RSC copyright 2021).

The diverse structure of terpenes makes them an excellent source of complex cyclic carbonates, as opposed to other bio-based feedstocks. The use of waste CO_2_ as a coupling agent after oxidation is a straightforward process. Recently, the synthesis of cyclic carbonates from terpenes has gained prominence in research. While past efforts focused on preparing five- and six-membered cyclic carbonates, there is now a growing interest in developing more complex compounds.^82^ It is thermodynamically efficient to react CO_2_ with compounds that have high free energy. In nature, fir cone oil and citrus fruits produce cyclic terpenes like limonene (both isomers). (*R*)-(+)-Limonene can be cost-effectively obtained from orange peel waste. The presence of several double bonds allows the epoxidation of 1,2-limonene oxide (1,2-LO), 8,9-limonene oxide (8,9-LO), and limonene dioxide (LDO). Coupling these compounds with CO_2_ results in the formation of corresponding carbonates (Fig. 5).

**Fig. 6:**
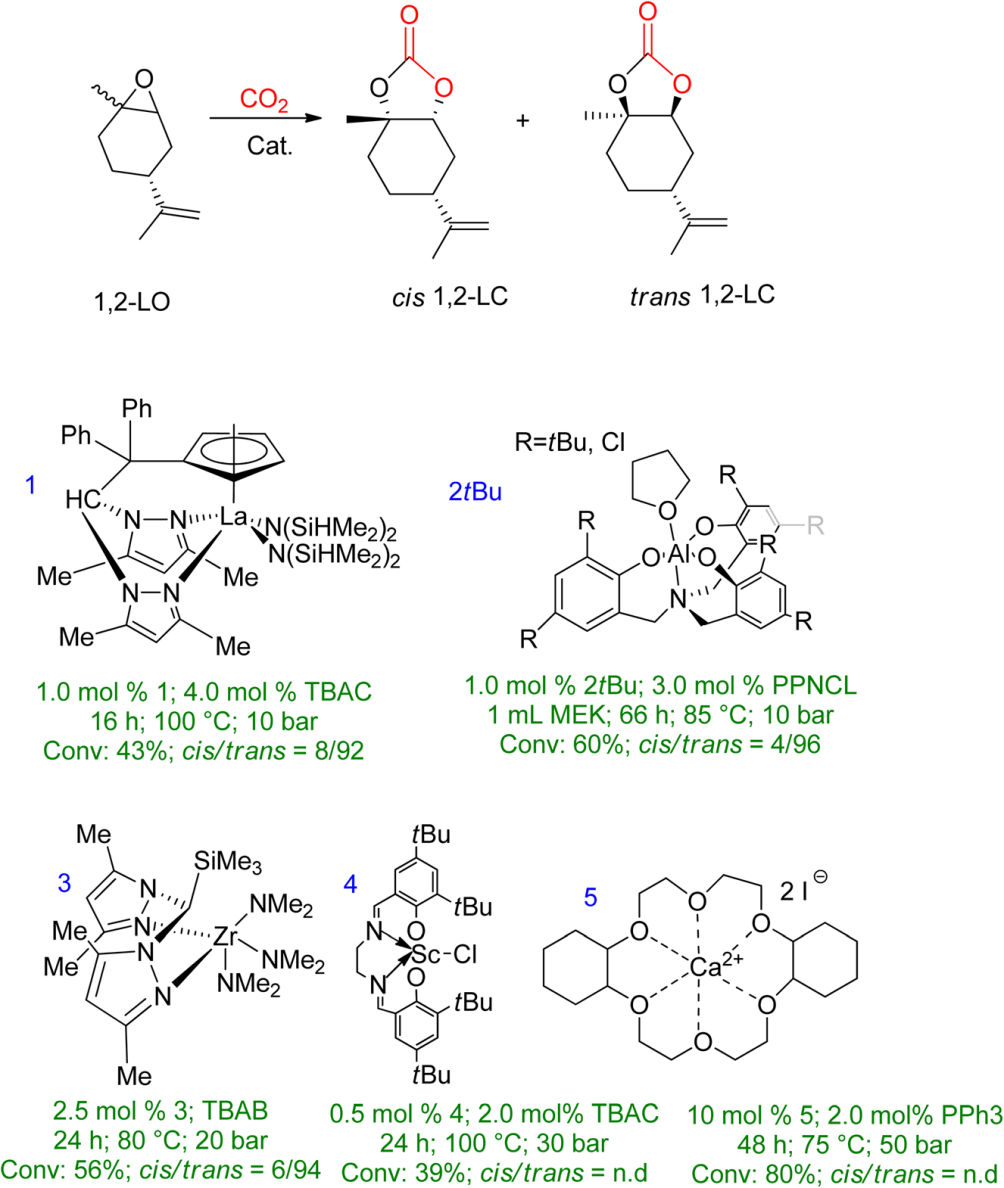
A metal-based catalytic system reported for the synthesis of 1,2-LC, along with comparisons of the reaction conditions and chemistry of the products (n.d. = no determination) (reproduced from ref. 82 with permission from RSC copyright 2021).

Extensive studies have been conducted on 1,2-LO due to its wide availability. However, when comparing sterically demanding 1,2-LO with other epoxides, the former exhibits lower reactivity during coupling. As a result, a longer reaction time (16–66 h) is required at higher temperatures and pressures (75–100 °C, 10–50 bar). Fig. 6 summarises metal-based catalytic systems that have been reported for the coupling of 1,2-LO and CO_2_.^83–87^

Several catalyst systems, including 1, 2*t*Bu, and 3, have been reported to selectively form *trans*-1,2-LC, leading to different reactivity between the *cis* and *trans* isomers of 1,2-LO.^83,84,86^ In 2016, efficient conversion of various terpene-based substrates was achieved using 2^R^/PPNCL, which produced LDO with good yield. However, the synthesis of LDC was unsuccessful due to the formation of polyether (PE) side products. The reaction conditions for LDO synthesis were 1.0 mol% 2R, 3.0 mol% PPNCL, 1.0 mL MEK, 66 h, 85 °C, *p*(CO_2_) = 1 MPa.^84^ Another study reported the formation of LDC with a concentration of up to 78% in the presence of 10.0 mol% 5/2.0 mol% PPh_3_ catalyst, after 48 h at 75 °C and 50 bar.^85^

Tetrabutylammonium halide (TBAX) is an efficient and effective catalyst for synthesizing 5-ring cyclic carbonates.^88^ Therefore, a similar reaction was carried out using TBAX as a catalyst for the reaction between 1,2-LO and CO_2_ with the substrate being ct-LO [43 : 57].^89^ The reaction conditions were extreme with high temperature, pressure, and reaction time (100 °C, 3 MPa, 20 h) and the catalyst consisted of 10 mol% ct-LO without any solvent. Tetrabutylammonium chloride (TBAC) had a 51% conversion rate of *cis*–trans-LO, with the individual conversion of *trans*-LO and *cis*-LO being 76% and 19%, respectively. However, the conversion rate and NMR yield decreased when tetrabutylammonium bromide (TBAB) and tetrabutylammonium iodide (TBAI) were used as activating catalysts. The order of catalytic activity increment was reported as TBAX > TBAB > TBAI < TBAC.

The research group continued their investigation using TBAC as the catalyst model to study the effect of reaction conditions. The typical conditions used were a 10 mol% catalyst based on *cis*–*trans*-LO, 20 h reaction time, 100 °C temperature, and 3 MPa of CO_2_ pressure. The study focused on varying reaction times, CO_2_ pressure, and the amount of TBAC. Prolonging the reaction time did not improve the conversion of *cis*–*trans*-LO, as 48 h and 72 h resulted in 65% and 69% yields, respectively. The next step was to vary the CO_2_ pressure while keeping all other parameters constant. The results showed that only increasing the pressure by 5 MPa increased the individual yield/conversion to *cis*-LO/*trans*-LO to 27 : 85. Finally, increasing the amount of TBAC increased *trans*-LO yield, while *cis*-LO conversion remained low.

A detailed kinetic study of the coupling reaction between 1,2-LO and CO_2_ in the presence of TBAC was recently reported, which found that the *trans*-isomer exhibits greater reactivity. The reaction was determined to be a first-order reaction with respect to all reactants, and the Eyring equation was used to determine the thermodynamic parameters of conversion. The entropy value was found to be 60.6 kJ mol^−1^ and the enthalpy value was −103.6 J (mol K)^−1^.^90^ In another recent study by Mikšovsky *et al.*,^91^ tetrabutylammonium-based halides and 1-ethyl-3-methyl imidazolium halides were used as homogeneous catalysts, while a supported ionic liquid was used as a heterogeneous catalyst for the synthesis of cyclic carbonates from limonene epoxides. It was found that imidazolium-based liquids have less catalytic activity in comparison or are even inactive when LO (*cis*/*trans*-mixture) is used as a substrate. TBAC proved to be the most selective and highest-yielding catalyst for converting the diastereomeric mixture (*cis*/*trans* = 44/56). Silica without immobilization, TBAC, and TBAB showed lower yield, while in the case of imidazolium-based catalysts TBAI yield increased, but the overall selectivity was still low. The same order of catalytic activity was seen for supported ionic liquid phases and ammonium-based ionic liquids (ILs) physisorbed on silica as it was for homogeneous mode.

**Scheme 5:**
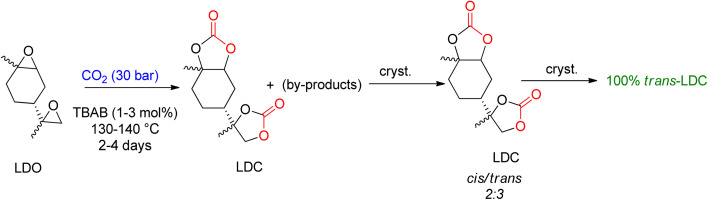
Formation of limonene di-carbonate (LDC) by the reaction of limonene di-oxide (LDO) and CO_2_ catalyzed by TBAB and subsequent crystallization to obtain *trans*-LDC (reproduced from ref. 82 with permission from RSC copyright 2021).

Mülhaupt's research group used LDC to prepare poly-hydroxy urethanes (Scheme 5).^92^ For the synthesis of LDC on a one-kilogram scale, TBAB was used, and the reaction parameters were optimized to allow for 3 mol% TBAB usage in less than 50 h to achieve complete LDO conversion. The resulting product was a brownish oil, which can be directly used to prepare non-isocyanate polyurethanes (NIPUs). Several by-products were identified after analyzing the reaction. LDC was cooled and crystallised to create a pure product, resulting in a 3 : 1 combination (*cis*-to-*trans* ratio). X-ray analysis proved that the *trans*-LDC produced by further crystallisation was 100% pure.^93^

**Scheme 6:**
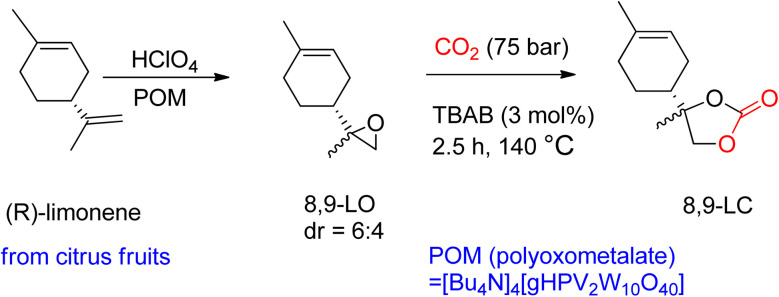
Highly selective (*R*)-(+)-limonene epoxidation to obtain 8,9-limonene oxide (8,9-LO) and subsequent reaction with CO_2_ to obtain 8,9-LC by TBAB-catalysed reaction (reproduced from ref. 82 with permission from RSC copyright 2021).

Through TBAB-mediated reaction, 8,9-LC was formed by reacting CO_2_ with 8,9-LO. To synthesize the desired carbonates, the less hindered terminal alkene of (*R*)-(+)-limonene was selectively epoxidized (Scheme 6). Perchloric acid was used as the oxidant, and bulky polyoxometalates (POM) were used as the catalyst. Compared to 1,2-LO, 8,9-LC is easier to produce due to less steric hindrance, reaching maximum conversion in only 2.5 h under comparable reaction conditions. Additionally, the two diastereomer-isomers obtained showed the same reactivity during coupling with CO_2_, which is in contrast to 1,2-LO, where *trans*-1,2-LO was found to be more reactive.^94^

### 2.3 Glycerol carbonate

Glycerol carbonate (GC), also referred to as 4-hydroxymethyl-1,3-dioxolan-2-one, is a bio-derived 5-membered ring carbonate synthesized from glycerol. It is one of the few newly commercialized bio-derived products from biomass.

#### 2.3.1 Glycerol carbonate synthesis from glycerol and CO_2_

**Scheme 7:**
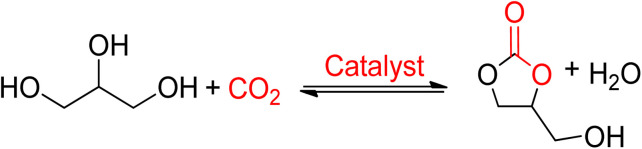
Glycerol carbonate (GC) obtained from glycerol and CO_2_ (reproduced from ref. 82 with permission from RSC copyright 2021).

Glycerol carbonate (GC) is a 5-membered ring carbonate synthesized from glycerol and is an example of a new bio-derived product from biomass that has been commercialized recently. The most suitable reaction is the direct reaction of glycerol with CO_2_, which produces solely the required product and water. This reaction is completely green and combines two waste reactants, as indicated in Scheme 7. Due to thermodynamic limitations and the requirement to identify a suitable dehydrating agent to remove water from the reaction, this reaction is not feasible for industrial production.^95^

**Scheme 8:**
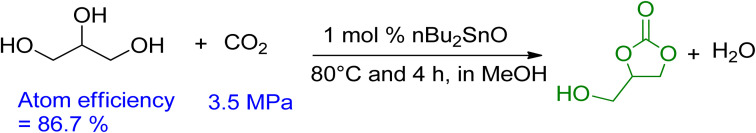
Improved carbonation of glycerol using MeOH as solvent.^98^

Vieville *et al.* reported the first attempt to synthesise GC from glycerol and CO_2_ in 1998.^96^ The reaction did not occur under supercritical CO_2_ conditions. The first successful production of GC was reported by Aresta *et al.* in 2006 using Sn-based catalysts under 5 MPa and 180 °C of pressure and temperature, respectively.^97^ A maximum yield of 5.7% was achieved without the use of a solvent, and molecular sieves were used for the removal of *in situ* water. During the mechanistic study, it was proposed that the Sn-based catalyst and CO_2_ could be inserted into glycerol by forming the active Sn-glycerate intermediate. George *et al.* intensified this process in 2009 using MeOH as a medium, increasing the yield of GC to 35%, as shown in Scheme 8.^98^ Methanol acted as a solvent and was involved in the reaction mechanism as well. Metal oxide/complexes, supported metal catalysts, modified zeolites, or hydrotalcite have been reported as catalytic systems. For GC synthesis, most heterogeneous catalysts require CO_2_ at high pressures and temperatures above 40 bar and 150 °C, respectively.

**Scheme 9:**
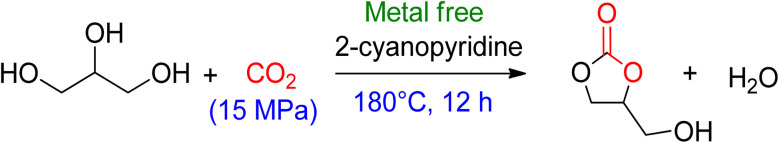
Metal-free formation of GC using 2-cyanopyridine (reproduced from ref. 82 with permission from RSC copyright 2021).

**Scheme 10:**
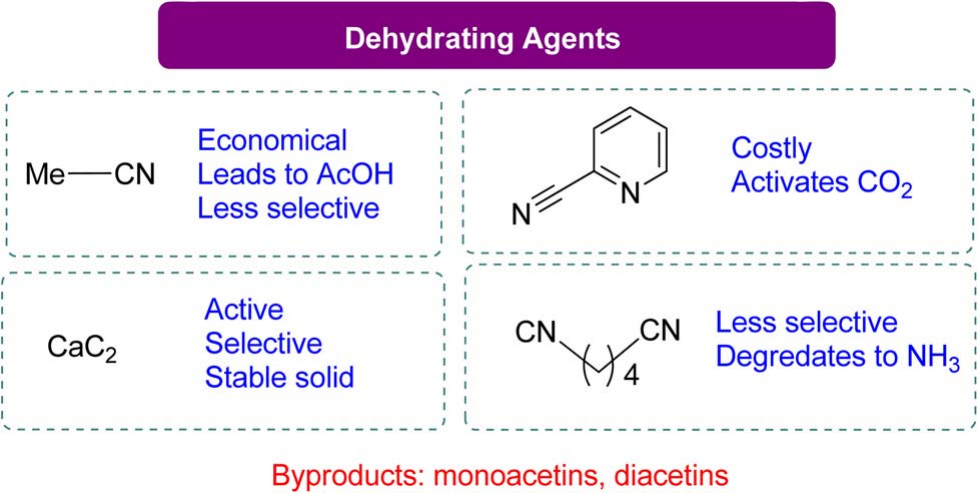
Comparison of different dehydrating agents in the formation of GC from glycerol and CO_2_ (ref. 18, 21, 99 and 100) (reproduced from ref. 82 with permission from RSC copyright 2021).

The dehydrating reagent for the removal of water was more efficacious than using molecular sieves. However, in some cases, a decrease in chemo selectivity towards GC was noted. The most used dehydrating agents for this reaction are acetonitrile, adiponitrile, and 2-cyanopyridine, as shown in Scheme 10. In most studies, acetonitrile is the first choice because it is cheap, but upon reaction with the water molecule, it generates acetamide *in situ*. The produced acetamide reacts with another water molecule to produce acetic acid, which decreases selectivity by forming mono- and di-acetins. McGregor *et al.*^18^ used adiponitrile as a dehydrating agent to remove water and found that it degrades to ammonia upon reacting with glycerol, resulting in a mixture of two regio-isomers of 4-HMO (4-(hydroxymethyl)oxazolidine-2-one). In 2016, Liu *et al.*^99^ employed 2-cyanopyridine as a highly effective dehydrating reagent. By using 3 equivalents of 2-cyanopyridine with a CeO_2_ catalyst in DMF at 4 MPa of CO_2_ and 150 °C, GC yields can increase to 79%. Recent research by Zhang *et al.*^100^ has shown the benefits of employing CaC_2_ as a dehydration agent for GC production. In their work, CaC_2_ was combined with Zn(OTf)_2_/phen (1,10-phenanthroline) in *N*-methyl-2-pyrrolidone (NMP), and the reaction was carried out at 50 bar CO_2_ pressure at 180 °C for 24 h to get 88% GC. In their experiment, CaC_2_ in combination with Zn(OTf)_2_/phen (1,10-phenanthroline) in NMP (*N*-methyl-2-pyrrolidone) at 50 bar CO_2_ pressure and 180 °C for 24 h yielded 88% GC. Su *et al.*^101^ reported a year later that GC can also be produced using 2-cyanopyridine at 15 MPa of CO_2_ and 180 °C without the use of a metal catalyst, with a 19% yield (Scheme 9). FTIR analysis confirmed the formation of a 5-membered ring *via* the activation of CO_2_ by 2-cyanopyridine, followed by a reaction with glycerol to form GC. The formation of this heterocycle was also supported by theoretical analysis, and glycerol was subsequently transesterified by it. Notably, there was no substantial GC production when using acetonitrile, 4-cyanopyridine, or 3-cyanopyridine (Table 3).

**Table 3:** Preparation of glycerol carbonate (GC) using dehydrating agents

System	Catalyst	Dehydrating agent	Pressure (MPa)	*T* (°C)	Time req.	Yield^a^/conversion^b^	Other reported	Reference
Homogeneous	*n*-Bu_2_Sn (OMe)_2_	Molecular sieves	5	180		5.7^a^	No solvent	97
Homogeneous	*n*Bu_2_SnO	13X-soda zeolites	3.5	80	4 h	35^a^	MeOH as medium	98
Heterogeneous	La_2_O_3_	Adiponitrile	4.5	160	18 h	58^b^		18
Heterogeneous	CeO_2_	2-Cyanopyridine	4	150		33.3^a^/38^b^		99
Heterogeneous	Zn (OTf)_2_/phen (1,10-phenanthroline)	CaC_2_	50 bars	180	24 h	88^a^	In NMP (*N*-methyl-2-pyrrolidone)	100
Metal-free	No	2-Cyanopyridine	15	180		18.7^a^/31.1^b^		101

a Yield.

b Conversion.

#### 2.3.2 Glycerol carbonate synthesis using coupling agents

**Scheme 11:**
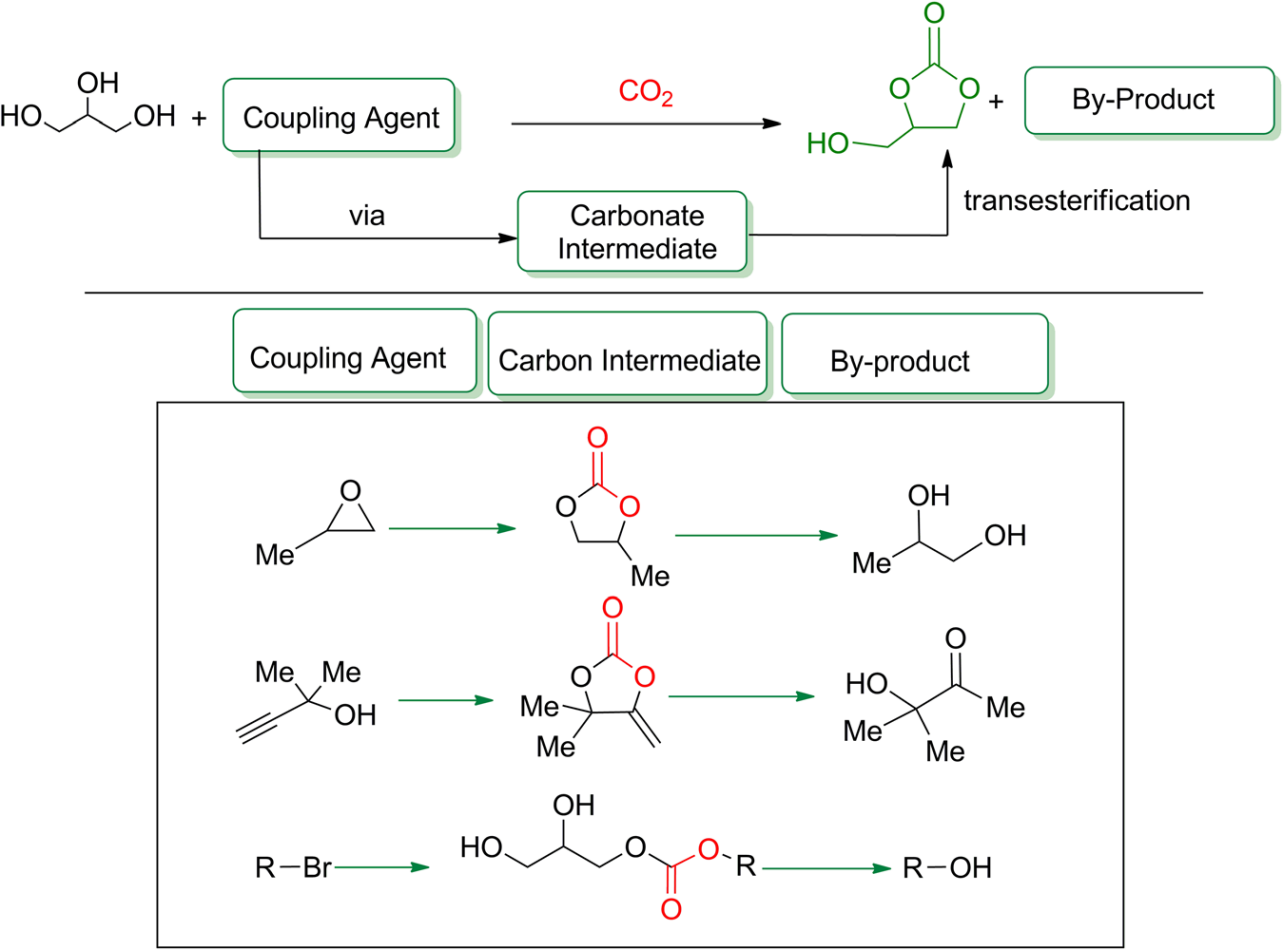
General reaction of glycerol with CO_2_ in the presence of a coupling agent, reproduced from (reproduced from ref. 82 with permission from RSC copyright 2021).

Glycerol carbonate (GC) can be effectively synthesized through the reaction of glycerol and CO_2_, facilitated by a coupling agent. This coupling agent enables the formation of a cyclic carbonate intermediate in situ, which facilitates the transesterification process, resulting in the formation of glycerol carbonate along with a by-product (Scheme 11). By incorporating a third reactant, this approach overcomes the inherent thermodynamic constraints of the direct glycerol-CO_2_ reaction and generates an additional by-product derived from this supplementary component. In 2012, Ma *et al.*^102^ (entry 1, Table 4) utilized propylene oxide (PO) as the first additive in the one-pot reaction of CO_2_ and glycerol. The substance with a hydroxyl group can act as a co-catalyst for halide-catalyzed reactions, promoting the reaction by hydrogen bonding. Thus, no additional co-catalyst was required for this reaction. The catalyst activity was affected by the solvent, which is why KI demonstrated the highest activity. By using 0.18 mmol of KI, 10 mmol of glycerol, and 20 mmol of PO, a 75% yield of GC could be achieved under 115 °C and 2 MPa in 1.5 h. The catalysts facilitate the formation of PC, which reacts with glycerol and undergoes *trans*-esterification to produce glycerol and propylene glycol. In 2018, Song *et al.*^103^ (entry 2, Table 4) developed P-DVB-(vIm-BuBr), a functionalized heterogeneous catalyst by introducing ionic liquid into the structure of DVB-based polymers. The catalyst was highly active, inexpensive, and had outstanding stability, making it suitable for industrial-scale use. When evaluating heterogeneous catalysts, reusability is critical. In addition to increasing the yield to 80%, the catalysts could be reused up to five times. By using 4 wt% catalyst loading of P-DVB-(vIm-BuBr), 25 mmol of glycerol, and 100 mmol of PO, a 75% yield of GC could be achieved under 100 °C and 2 MPa in 4 h.

**Table 4:** Preparation of GC using coupling agents (reproduced from ref. 82 with permission from RSC copyright 2021)

System	Catalyst	Coupling agents	*P* (MPa)	*T* (°C)	Time req.	Yield%	Reference
Heterogeneous	KI	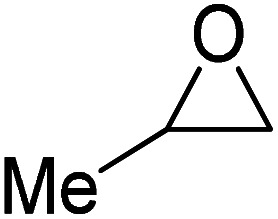	2.0	115	1.5 h	77	102
Heterogeneous	P-DVB-(vIm-BuBr)	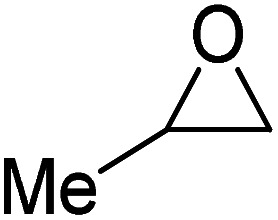	2.0	100	4 h	81	103
Heterogeneous	Ag_2_CO_3_/xanthophores	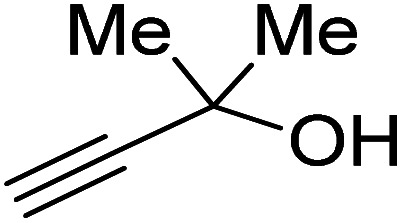	1.0	80	12 h	82	104
Heterogeneous	Silver sulfadiazine and Et_4_NBr	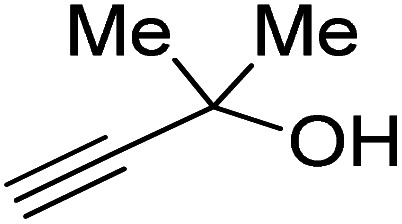	0.1	80	24 h	56	105
Heterogeneous	Amidine-CO_2_ adduct/MTBD	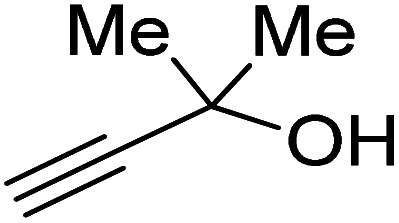	0.1	25	24 h	85	106
Heterogeneous	DBU (8-diazabicyclo[5.4.0]undec-7-ene), DMF	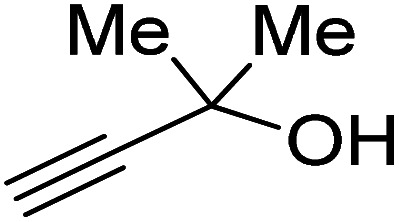	3.0	120	10 h	97	107
Heterogeneous	DBU (8-diazabicyclo[5.4.0]undec-7-ene), BmimPF_6_	CH_2_Br_2_	1.0	70	18 h	86	108
Heterogeneous	Guanidine catalyst	*n*-BuBr	0.1	50	4 h	74	109

Propargylic alcohol (dimethyl ethynyl carbinol) has been used as a coupling reagent along with PO. He *et al.*^104^ utilized Ag_2_CO_3_/xanthaphos catalyst in MeCN with 1 MPa CO_2_ pressure at 80 °C to produce a good yield of GC in one pot approach using CH_3_CN as a solvent (entry 3, Table 4). Li *et al*.^105^ improved the process by using silver sulfadiazine and the Et_4_NBr synergistic system without any solvent (entry 4, Table 4), but the yield of GC decreased. Silver sulfadiazine activated the hydroxyl groups simultaneously in propargylic alcohols (or vicinal diols), resulting in an excellent performance. The Lu and Liu groups reported GC preparation by organo-catalytic approach. Zhou *et al.*^106^ used N-hetrocyclic carbene-CO_2_ adducts which formed an intermediate and further transesterification took place with glycerol catalyzed by MTBD (methyl-1,5,7-triazabicyclo [4.4.0] dec-5-ene) to achieve an 85% yield of GC at room temperature and 0.1 MPa (entry 5, Table 4). Han *et al.*^107^ reported a three-component reaction using DBU (8-diazabicyclo [5.4.0] under-7-ene) to promote both steps, resulting in 97% yield of GC at 120 °C and 3 MPa in 10 h (entry 6, Table 4). Alkyl halides can also be used as effective reactants, but they generate halogen-containing waste, making the method unsustainable. Jang *et al.*^108^ reported the use of DBU, BmimPF_6_, and CH_2_Br_2_ for direct coupling of various alcohols, including glycerol, without using any Mitsunobu-type reagents (entry 7, Table 4). At 70 °C and 1 MPa, it was possible to achieve 86% yield. Mihara *et al.*^109^ used *tert*-butyl tetramethyl guanidine and *n*-BuBr as an additional component at 50 °C and 0.1 MPa CO_2_ pressure employing NMP (*N*-methyl pyrrolidone) as a solvent to obtain GC in 74% yield (entry 8, Table 4). The authors suggest that the ionic liquid is effective in improving CO_2_ solubility, glycidol (Gly) may react with an NHC carbene formed that can capture/activate CO_2_.

#### 2.3.3 Glycerol carbonate synthesis from glycidol

Glycidol (Gly), an epoxy alcohol derivative, can be converted into glycerol carbonate (GC) as a precursor, instead of using bio-glycerol. This waste product from epichlorohydrin synthesis can be recycled in two steps to produce GC with excellent atom efficiency and eco-friendliness.^110^ Many catalytical systems have been reported for CO_2_ cycloaddition to oxirane and glycidol (Gly). Gly has relatively high reactivity compared to PO and styrene oxide. Two ways in which non-innocent hydroxyl groups can actively participate in the reaction have been identified. A paramount progress in this field was reported in 2016 by Monica *et al.*^111^ using TBAB as a catalyst. Under optimal conditions, more than 99% conversion was afforded to GC. Furthermore, binary catalyst derived from Gly/TBAB was successfully tested on other epoxide/CO_2_ combinations, with significantly higher substrate conversions than without Gly.

Rintjema *et al.*^112^ discovered that the hydroxyl group on Gly, which can be used to create transitory hemi-carbonate species, can be used to activate CO_2_. Halide-free processing is made possible by this activation, which causes the oxirane ring to be opened by an intramolecular nucleophile under mild conditions and without the requirement for an external nucleophilic addition. It has been determined the exact process by which the oxirane ring of Gly is opened intramolecularly by the production of hemi-carbonate. Additionally, metal and halide-free production of GC using a packed bed reactor under flow conditions has been reported.^113^ Several catalytical systems, including heterogeneous, homogenous, and organo-metallic, have been reported to produce GC from Gly. However, not all catalytic systems are superior to simple TBAB.

#### 2.3.4 Glycerol carbonate synthesis using heterogeneous catalysts

**Scheme 12:**
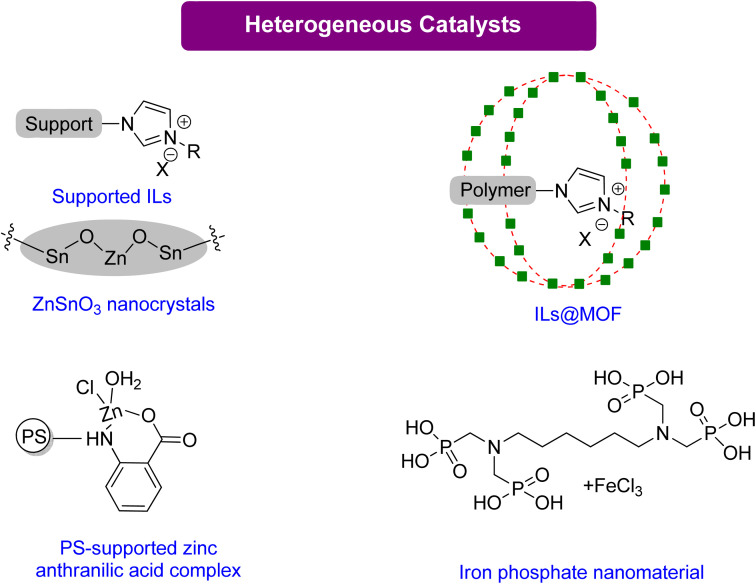
Highly efficient heterogeneous catalysts for the synthesis of GC from Gly,^120–123^ (reproduced from ref. 82 with permission from RSC copyright 2021).

Heterogeneous catalysts offer the advantage of easy recovery after post-synthesis processes such as product filtering or extraction, as illustrated in Scheme 12. Numerous heterogeneous systems used to synthesise GC from Gly are derived from ILs.^114–119^ The reaction occurs in two steps: firstly, the nucleophilic anion opens the oxirane ring, and secondly, an IL forms hydrogen bonds between the acidic C2 proton of the imidazolium ring and the oxirane ring.

Multifunctional components integrated into composite materials can enhance the activity for specific uses. Jiang *et al.*^120^ reported an efficient composite catalyst, polyILs@MIL-101, by confining imidazolium-based polyionic liquids (polyILs) into a metal–organic framework (MOF) material MIL-101 *via in situ* polymerizations of encapsulated monomers. This material has CO_2_-capturing capability and is a good catalyst without any co-catalyst under normal atmospheric pressure. With high surface area and hierarchical pores, PolyILs@MIL-101 is a promising catalyst material. The superior catalytic activity of polyILs@MIL-101, in comparison to either polyILs or MIL-101, is due to the synergy between the CO_2_ capturing capability, the Lewis base site in polyILs, and the Lewis acid site in the MOF. Only a few reports have explored the synergistic effects of CO_2_ capture and conversion. The results of this study demonstrate the necessity of enriching CO_2_ using a heterogeneous catalyst to assist in its conversion. Moreover, this paper proposes a strategy for designing practical catalysts that could be used in the future to directly capture and convert CO_2_ from flue gases.

Ghosh *et al.*^121^ reported using iron-phosphonate hybrid organic–inorganic nanoparticles HPFP-1(NP) as a nano-catalyst for synthesizing organic carbonate at mild conditions and CO_2_ under atmospheric pressure. This catalyst demonstrated excellent recyclability and reusability. In addition, a polystyrene–zinc–anthra complex was found to be an efficient catalyst, achieving 89% conversion to GC in 5 h.^122^ While both HPFP and PS-Zn require TBAB as a co-catalyst, zinc stannate nanocrystals do not need any co-catalyst. These nanocrystals could also be recovered and reused without losing their activity in the presence of PEG-600 as a green solvent, showing the potential of nano-catalysts as an environmentally friendly solution.^123^ The summary of the homogenous catalytical system reported for GC production from Gly is given in Table 5.

**Table 5:** Summary of the heterogeneous catalytical system based on ionic liquids for GC production from Gly

Catalyst	Pressure	Temperature (°C)	Time req.	Conversion^a^/yield^b^	Other reported	Reference
PolyILs@MIL-101	1.5 bar	70	24 h	>99%^a^	2 mL acetonitrile	120
PS-Zn-anthra complex	1 atm	25	4 h	89%^b^	Bu4NBr, TON/TOF = 141/28	122
Zinc-stannate nanocrystals	1 atm	80	10 h	91%^b^	PEG-600 as a green solvent	123

a Conversion.

b Yield.

#### 2.3.5 Glycerol carbonate synthesis using homogeneous catalysts

**Scheme 13:**
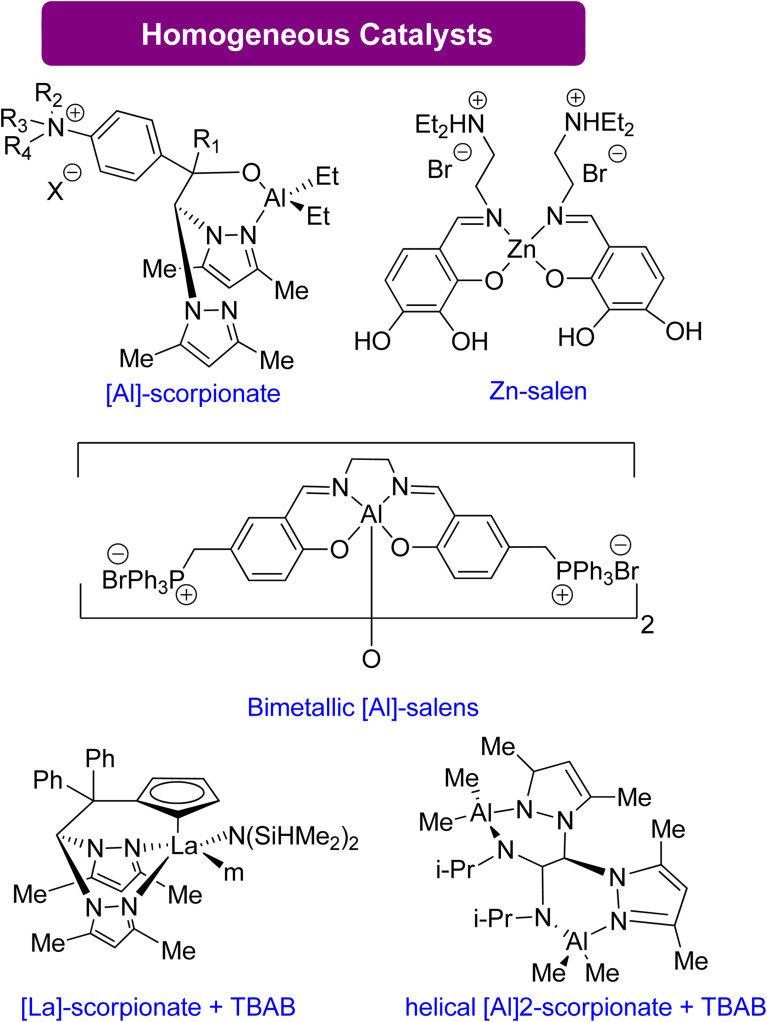
Synthesis of GC from Gly using highly effective homogeneous catalysts^83,124–129^ (reproduced from ref. 82 with permission from RSC copyright 2021).

Compared to their heterogeneous counterparts, homogeneous catalysts offer several advantages due to their use of earth-abundant materials and lower loading requirements. Halide ion-based bifunctional catalysts have shown promising results in terms of conversion and selectivity. Martínez *et al.*^124–126^ reported the use of aluminium scorpionate complexes (Scheme 13) as an efficient catalyst for the conversion of Gly to GC with low catalyst loading (0.25–0.5 mol%) at a moderate temperature of 70–85 °C. Similarly, He *et al.*^127^ obtained favourable results using a bifunctional Zn salen-like complex, achieving substantial activity with a low catalyst loading of 0.3 mol% at 0.1 MPa and 100 °C. Another bifunctional bimetallic aluminium salen complex was reported by North *et al.*,^128^ allowing for complete conversion at moderate conditions (0.1 MPa, 70 °C) in 3 h. Homogeneous metal catalysts combined with halides as co-catalysts have also exhibited high activity. Navarro *et al.*^129^ stated the use of an aluminium-based catalyst, while Martinez *et al.*^83^ reported lanthanum-based scorpionate catalysts. The La-based catalyst demonstrated conversion at a low catalyst loading of 0.05 mol%, achieving over 90% yield at moderate conditions (1 MPa, 70 °C) in 4 h. The summary of the homogenous catalytical system reported for GC production from Gly is given in Table 6.

**Table 6:** Summary of the homogenous catalytical system reported for GC production from glycidol (Gly)

Catalyst	Loading	Pressure (MPa)	Temperature (°C)	Time Req.	Co-catalyst	Reference
Aluminium scorpionate complexes	0.25–0.5 mol%	—	70–85	—	No co-catalyst	124–126
Bifunctional zinc salen-like complex	0.3 mol%	0.1	100	—	No co-catalyst	127
Bimetallic aluminium salen complexes	—	0.1	27	3 h	No co-catalyst	128
Heteroscorpionate lanthanum complex	0.05 mol%	1.0	70	4 h	Catalytic amount of halide as co-catalyst	83
Bimetallic helical aluminium complex	0.5 mol%	1.0	50	8 h	Catalytic amount of halide as co-catalyst	129

#### 2.3.6 Glycerol carbonate synthesis using organocatalysts

**Scheme 14:**
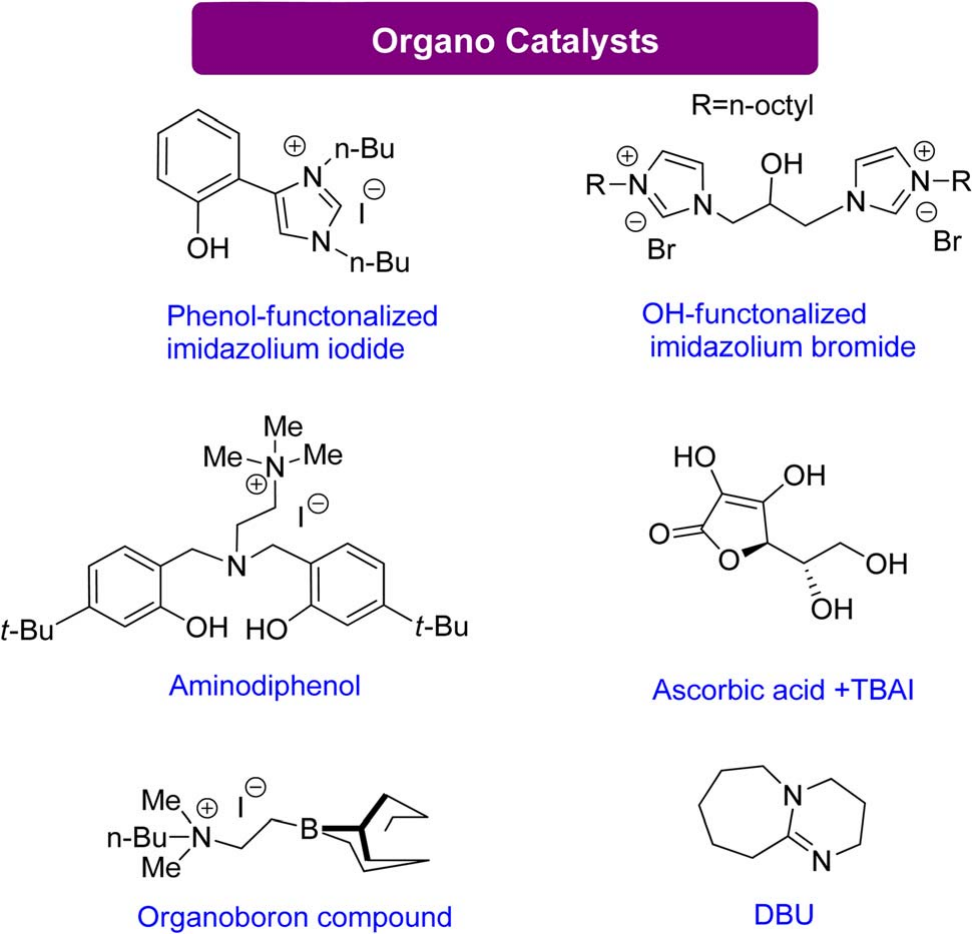
Efficient organo catalysts reported for the conversion of Gly into GC^130–135^ (reproduced from ref. 82 with permission from RSC copyright 2021).

Organocatalysts have shown activity analogous to metal-based catalysts in activating the oxirane ring. They achieve this by forming a hydrogen bond that facilitates ring opening by a nucleophile. Several organocatalytic compounds have been reported that are recyclable and efficient. For example, Anthofer *et al.*^130^ and Castro-Osma *et al.*^131^ reported an organocatalyst containing imidazolium rings and halide counter anions (Scheme 14), while Kim's group reported a scorpionate-type organocatalyst comprising an aminophenol scaffold and a quaternary ammonium salt.^132^ Yadav *et al.* reported ascorbic acid combined with a co-catalytic amount of halide TBAI that achieves high yields of GC at mild temperature and pressure conditions.^133^ Zhang *et al.* reported alterable organoboron bifunctional catalysts that are the most efficient catalysts known to date.^134^ These catalysts can withstand high temperatures of up to 150 °C and harsh conditions of 20 MPa CO_2_ and 120 °C. They achieve activation of the oxirane ring through a Lewis acid-like boron that activates the ring near the ammonium iodide unit.

Sopeña *et al.*^135^ concluded that DBU can enhance GC production from Gly and other epoxy alcohols by creating Hemi-carbonate intermediates in mild conditions. Furthermore, a new organocatalytic process has been discovered to couple tri- and tetra-substituted epoxides with CO_2_, allowing for the synthesis of cyclic carbonates previously difficult to produce under mild reaction conditions. This discovery is advantageous for industrial applications. By controlling the substrate conversion of CO_2_, new heterocyclic scaffolds can be created with enhanced synthetic potential.

## 3. New methods for the synthesis of cyclic carbonates

### 3.1 Synthesis of cyclic carbonates in flow reactors

The production of cyclic carbonates from epoxides and CO_2_ comprises a gas–liquid process in which mass is transferred from the gas phase to the liquid phase, followed by a catalytic cycloaddition reaction.^136^ The rate of CO_2_ mass transfer has been identified as a critical factor for controlling the reaction rate in batch reactors.^137^ An effective reactor design is required to attain high rates of heat and mass transfer. However, traditional batch reactors are less efficient, costly, and less secure. In contrast, continuous flow systems have several advantages, including lower reaction temperatures, shorter residence times, and ease of industrial scaling.^138^ As a result, continuous flow reactors can address many of the drawbacks of conventional batch reactors. Moreover, the design of the reactor significantly affects the gas–liquid reaction rates.^139^ Recent advancements in flow chemistry have enabled the chemical transformation and incorporation of CO_2_ into valuable organic compounds. These advances have made CO_2_ attractive to both industry and academia.^140–143^ Batch transformations using CO_2_ as a C1 synthon have rapidly developed,^144–147^ while CO_2_ reactions in continuous flow systems have become increasingly popular. Compared to conventional batch reactors, flow reactors provide several advantages. Firstly, they have a large surface-to-volume ratio, enabling effective mixing of different phases and precise temperature control. This rapid heat and mass transfer can greatly increase the effectiveness of reactions. Secondly, flow reactors can be much safer due to their excellent thermal management and low material hold-up, facilitating transformations that are difficult to perform in batch reactors, streamlining multistep processes, and optimizing reactions.^148–150^ Additionally, small flow reactors can produce large quantities of product because of their numbering-up methodology. Thus, lab research can be applied feasibly in the industrial setting within a short period.^151^

**Fig. 7:**
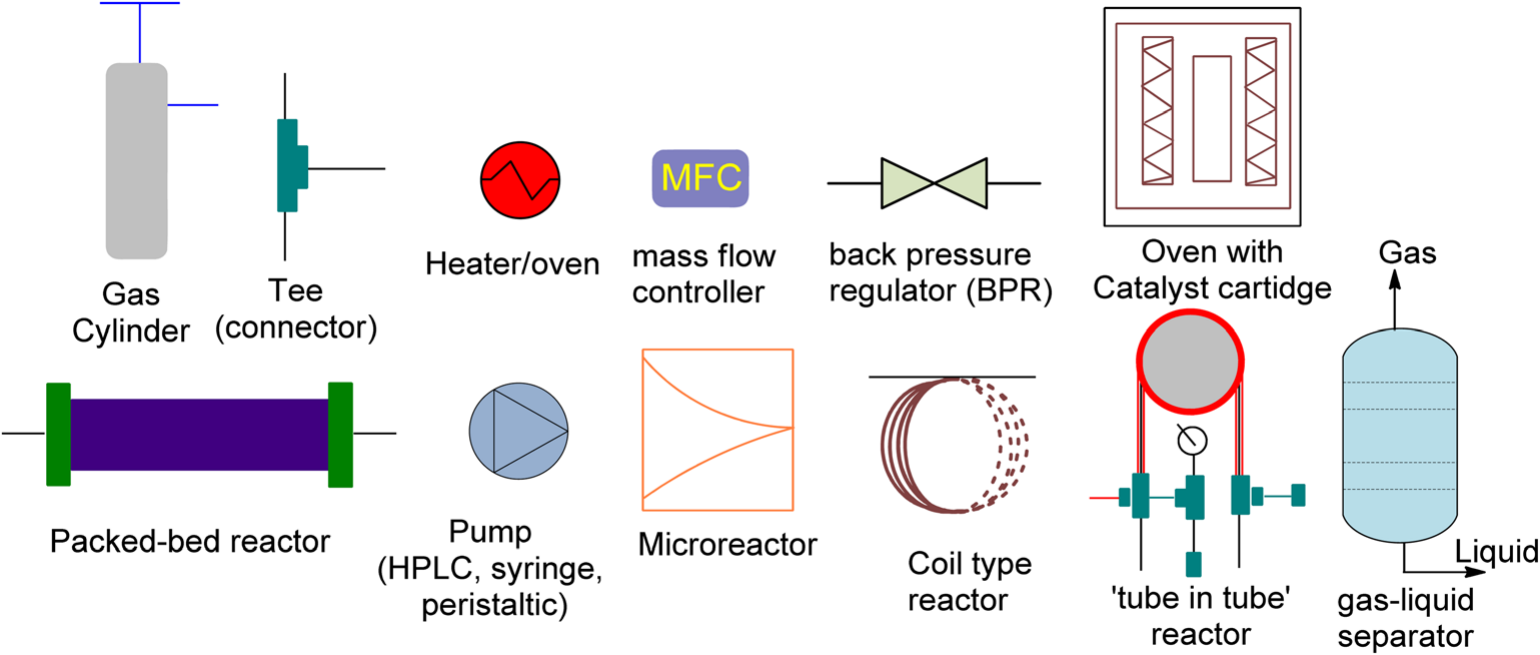
The schematic diagram for the representation of reaction equipment (reproduced from ref. 154 with permission from Wiley-VCH Verlag copyright 2021).

Although flow chemistry offers several advantages for CO_2_ transformation, its application is still a challenge. The low reactivity of CO_2_ makes many batch reactions slow, and different flow patterns can occur due to the reactor geometry and operating conditions.^152^ These patterns, such as annular flow, bubble flow, slug flow and churn flow, directly affect the experimental results. Thus, continuous processes require different operating parameters than batch processes, which must be adjusted accordingly. Additionally, CO_2_ fixation under flow conditions poses other challenges, such as product separation, catalyst recycling, and solvent and/or waste compatibility during multistep reactions. Innovative approaches have been reported to solve these issues, but further developments are necessary, including the development of new concepts, more efficient catalysts, and novel equipment. Process intensification has exciting possibilities and flow chemistry should be viewed as a complement to batch chemistry rather than as its competitor. Research and development efforts are needed for commercialization, and high-potential applications of process intensification include biological CO_2_ transformation, catalytic CO_2_ transformation, electrochemical CO_2_ transformation, and CO_2_ transformation using plasma technology.^153^ These advancements can significantly reduce the feasibility gap between newly developed sustainable technologies and previous technologies. Flow platforms are commonly equipped with the equipment listed in Fig. 7.

**Fig. 8:**
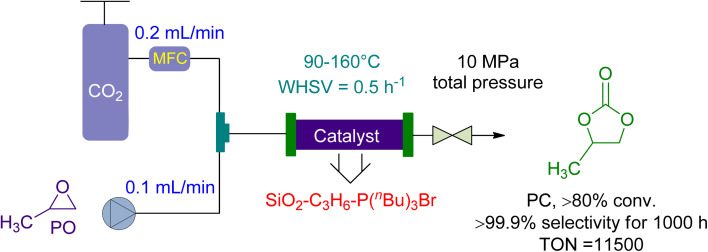
The catalytic coupling between Propylene oxide and CO_2_ catalysed by SiO_2_–C_3_H_6_–P(*n*-Bu)_3_Br (reproduced from ref. 156 with permission from RSC copyright 2017).

Ionic liquids have become a popular catalysis method in recent years due to their stability, non-flammability, recyclability, and tailoring ease.^137,155^ The cycloaddition of CO_2_ and propylene oxide (PO) to create PC was reported on by Takahashi *et al.* in 2006.^156^ They used a packed-bed reactor with an effective organic–inorganic hybrid catalyst. Compared to homogeneously used onium-salts, immobilized phosphonium-halides increased catalytic activity significantly. The inorganic-acid support also synergistically enhanced the organic component (Fig. 8). SiO_2_–C_3_H_6_–P (*n*-Bu)_3_ I had a pseudo-first-order rate constant 300 times greater than P (*n*-Bu)_4_I, normalized to phosphorus atoms. The fixed bed flow reactor used 10 g of SiO_2_–C_3_H_6_–P(*n*-Bu)_3_Br as a catalyst, with CO_2_ and PO flow rates of 0.1 and 0.2 mL min^−1^, respectively. Under 10 MPa, the temperature increased from 90 to 160 °C, yielding 80% in over 1000 h with 99% selectivity. One phosphonium group can produce more than 11 500 molecules of PC. Using packed-bed reactors and supported catalysts, this ground-breaking study found that the cycloaddition of CO_2_ to PC could be accomplished with remarkable efficiency.

**Fig. 9:**
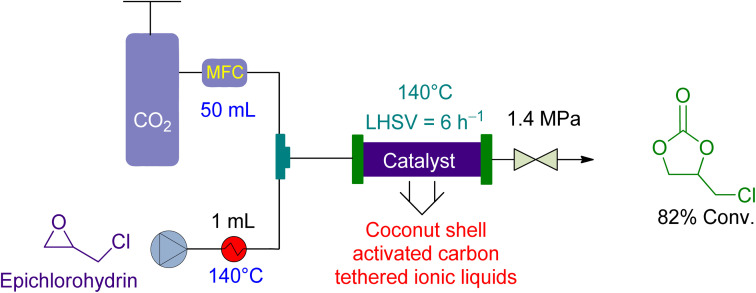
The catalytic coupling of epoxide and CO_2_ using coconut shell activated carbon (CSAC) tethered ionic liquid as a catalyst.^157^

Zhang *et al.*^157^ examined the utilisation of a packed-bed reactor for the cycloaddition of epichlorohydrin in 2015. They used coconut shell activated carbon (CSAC) anchored ionic liquids as a catalyst and no solvent (Fig. 9). The epichlorohydrin flowrate was maintained at 1.0 mL min^−1^ and the CO_2_ flowrate at 50 mL min^−1^. The reaction was carried out at 1.4 MPa and 140 °C, resulting in an 82% yield in 5 h, with a liquid hourly space velocity (LHSV) of 6 h^−1^.

**Fig. 10:**
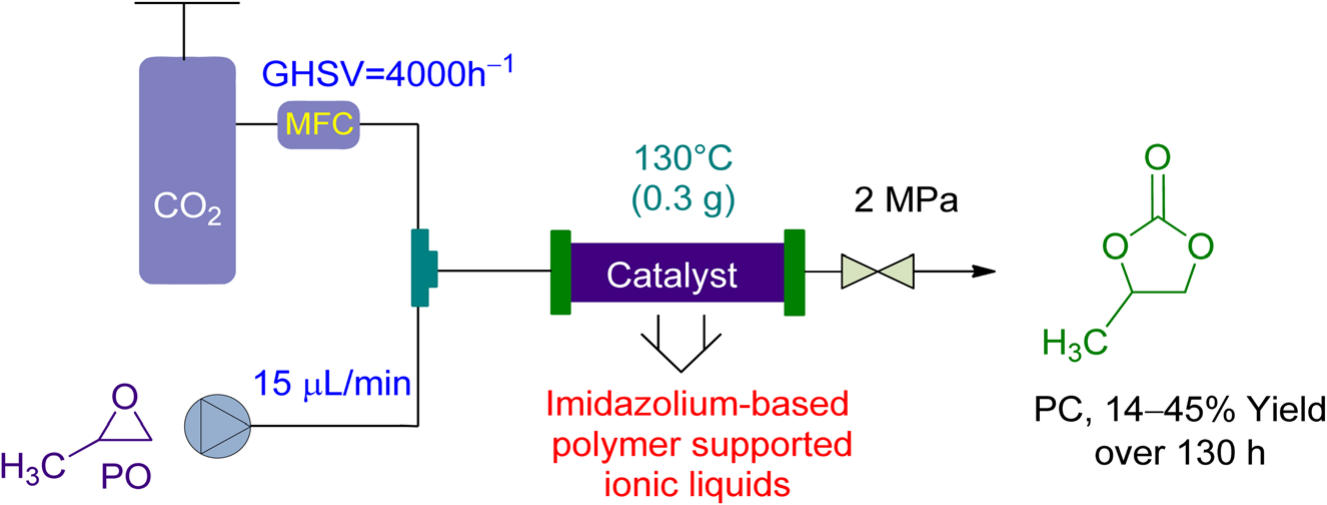
The catalytic coupling between Propylene oxide and CO_2_ catalysed by Polymer supported ionic liquid (PSIL) (reproduced from ref. 158 with permission from RSC copyright 2017).

In 2017, Wang *et al.*^158^ utilized a fixed-bed reactor to perform the cycloaddition of CO_2_ to PO to PC using newly synthesized PSIL(IMD) Imidazolium-based polymer-supported ionic liquids as a catalyst, without the use of a solvent. A total of 0.3 g of the catalyst was used in the experiment (Fig. 10). The gas hourly space velocity (GHSV) of the PO was kept constant at 4000 h^−1^ while the flow rate was kept at 15 μL min^−1^, under a 2 MPa pressure. With a liquid hourly space velocity (LHSV) of 6 h^−1^, a yield of 41 to 45% was attained in 130 hours. This system demonstrated good activity and was a significant milestone in the field.

**Fig. 11:**
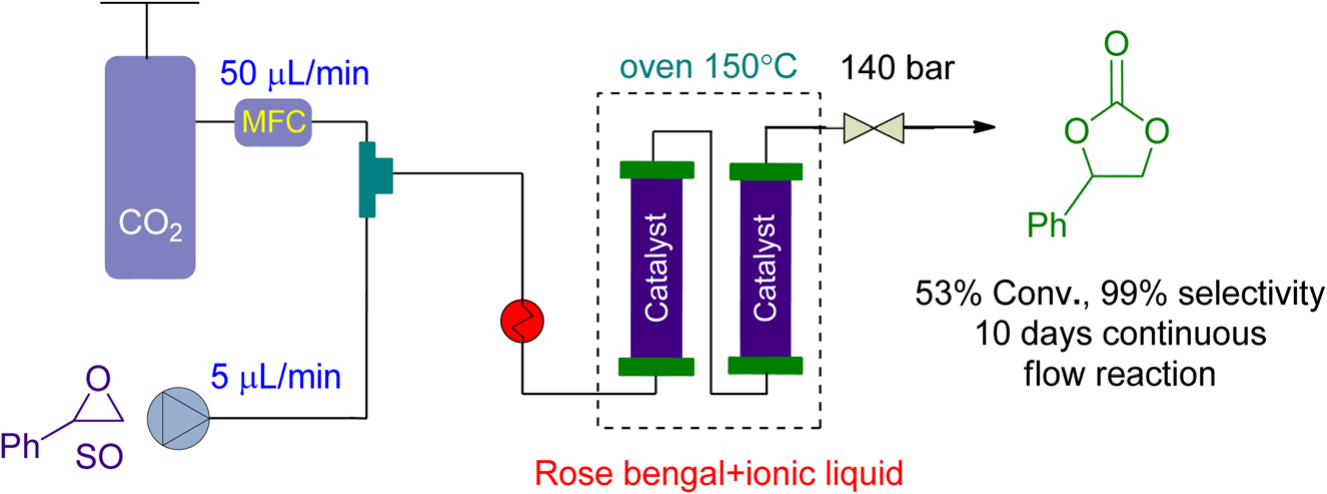
The catalytic coupling between styrene oxide and CO_2_ catalysed by Rose Bengal (RB) based polymer (reproduced from ref. 159 with permission from ACS, copyright 2021).

For CO_2_ cycloaddition to epoxides in a continuous flow reactor, Valverde *et al.*^159^ looked into the use of multifunctional polymers based on ionic liquids and Rose Bengal (RB) fragments (Fig. 11). A highly effective catalyst was produced under flow conditions by modifying the RB characteristics by changing the chemical nature of the supporting liquid. The substrate and CO_2_ flowed at rates of 5 and 50 μL min^−1^, respectively. At 140 bar and 150 °C, the system achieved 53% conversion over 10 days, with no leaching and no decrease in catalytic activity.

**Fig. 12:**
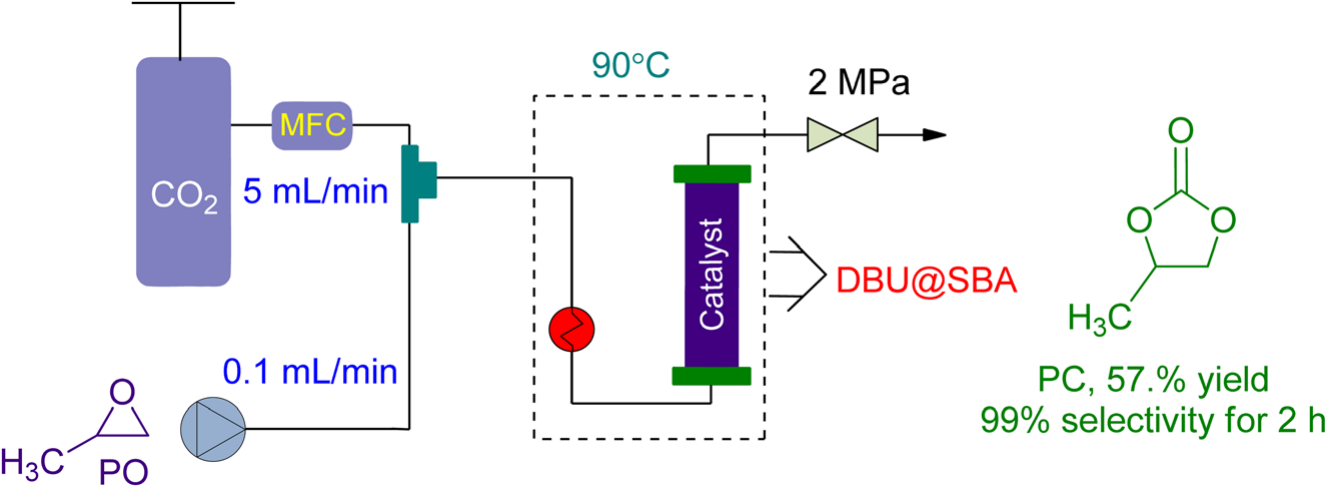
The coupling of propylene oxide with CO_2_ catalysed by DBU@SBA-15 (reproduced from ref. 160 with permission from Elsevier copyright 2021).

In 2021, Yin *et al.*^160^ investigated the use of a green DBU-based IL catalyst (DBU@SBA) for CO_2_ fixation and PC synthesis in a new packed bed reactor design (Fig. 12). The reactor had a substrate flow rate of 0.1 mL min^−1^ and CO_2_ flowrate of 5 mL min^−1^. At 2 MPa and 90 °C, the reaction was conducted, yielding 57.1% conversion after 2 h and 16.86% after 24 h of continuous operation. However, the catalytic activity was lost after 26 h, resulting in only a 4% yield.

**Fig. 13:**
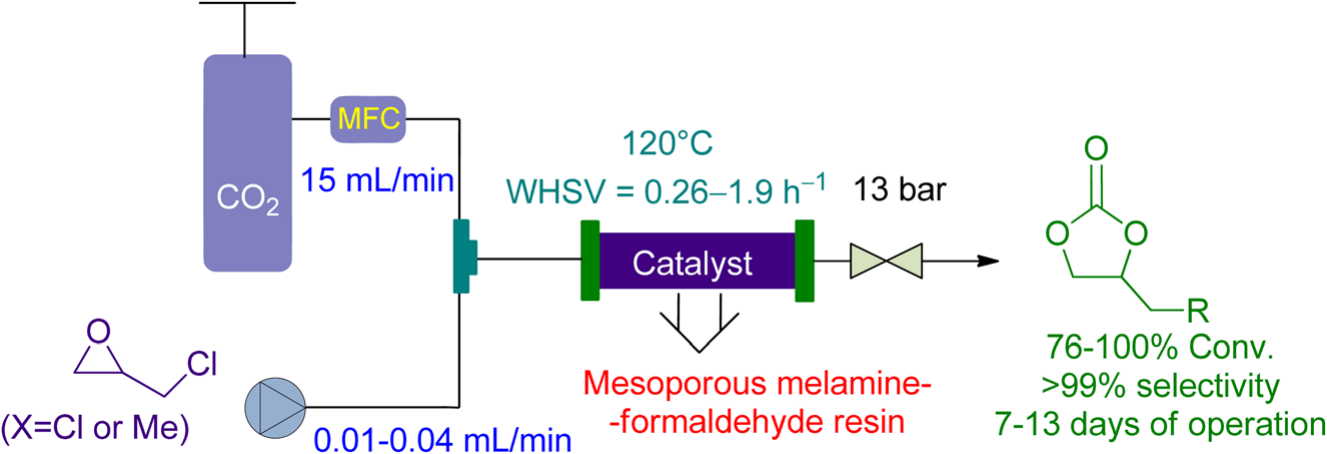
Mesoporous melamine-formaldehyde resins (MFFR) catalysed epoxide-CO_2_ coupling (reproduced from ref. 161 with permission from ACS, copyright 2020).

Bui *et al.*^161^ reported the use of a vertical-fixed bed reactor for the cycloaddition of CO_2_ to epichlorohydrin and 1,2 butylene oxide using economical Mesoporous melamine-formaldehyde resins (MMFR) as a heterogeneous catalyst (Fig. 13).

### 3.2 Synthesis of cyclic carbonates in microflow reactors

**Fig. 14:**
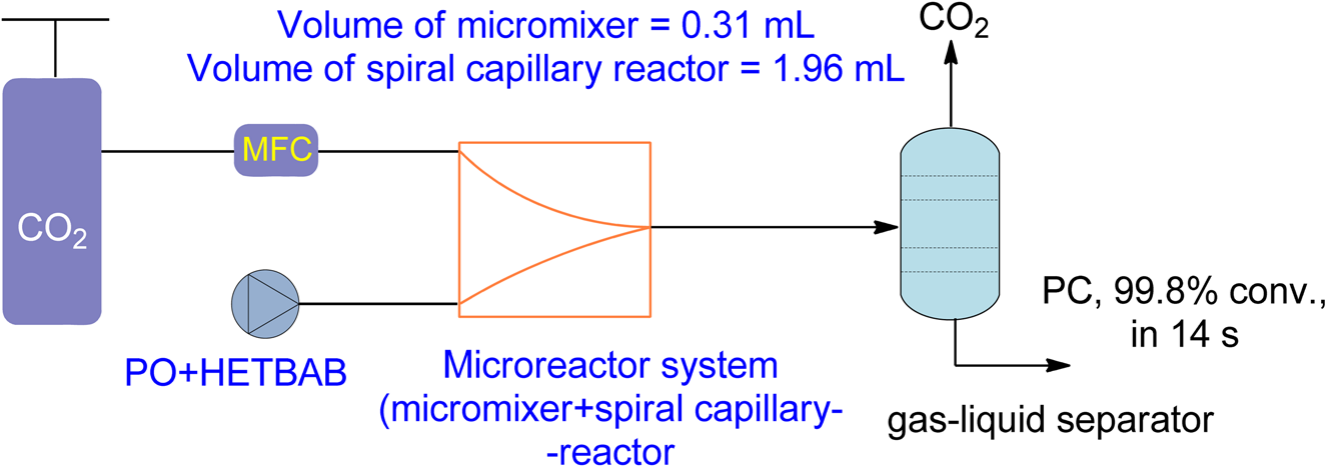
2-Hydroxyl-ethyl-tributyl ammonium bromide (HETBAB) catalysed reaction in micro reaction system including micromixer and capillary reactor (reproduced from ref. 136 with permission from RSC copyright 2013).

New types of flow reactors have been developed to improve the efficiency of production. Microreactors are ideal for two-phase gas–liquid processes due to their controllability and large specific surface area. Moreover, they offer opportunities for homogeneous catalysis for CO_2_-based chemical reactions.^162,163^ Zhao *et al.*^136^ investigated the use of a microreactor for the cycloaddition of PO to PC using a hydroxyl functionalized ionic liquid (HETBAB) as a catalyst. The high surface-to-volume ratio of the microreactor resulted in a significant improvement in heat and mass transfer compared to a conventional CSTR (Fig. 14). The reaction achieved a yield of 99.8% in just 14 seconds at 180 °C and 3.0 MPa.

**Fig. 15:**
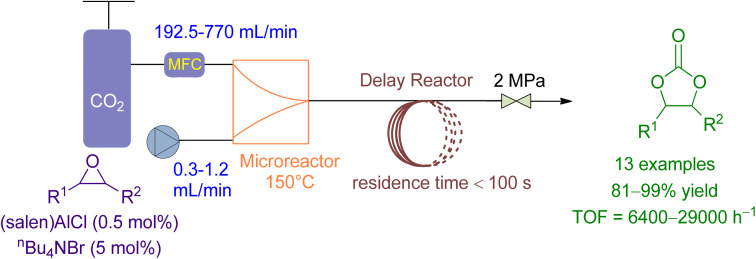
Coupling reaction of CO_2_ in the presence of (Salen) AlCl (reproduced from ref. 164 with permission from RSC copyright 2018).

Li *et al.*^164^ reported the use of a microreactor followed by a delayed reactor for the cycloaddition of CO_2_ to various epoxides using a binary catalytic system (Fig. 15). The flowrate of epoxide and CO_2_ was maintained at 0.3–1.2 mL min^−1^ and 195.2–770 mL min^−1^, respectively, achieving 90 to 99% conversion in less than 100 seconds with TOF ranging from 6800 to 14 700 under 2.0 MPa and 150 °C. Compared to a conventional stirred reactor, the microreactor reaction exhibited significantly higher TOF values, a higher and stable reaction rate, and negligible effect by the CO_2_/epoxide molar ratio. Furthermore, the “Electrophile–Nucleophile” synergistic effect in the microreactor could be enhanced to further improve the catalytic activity.

**Fig. 16:**
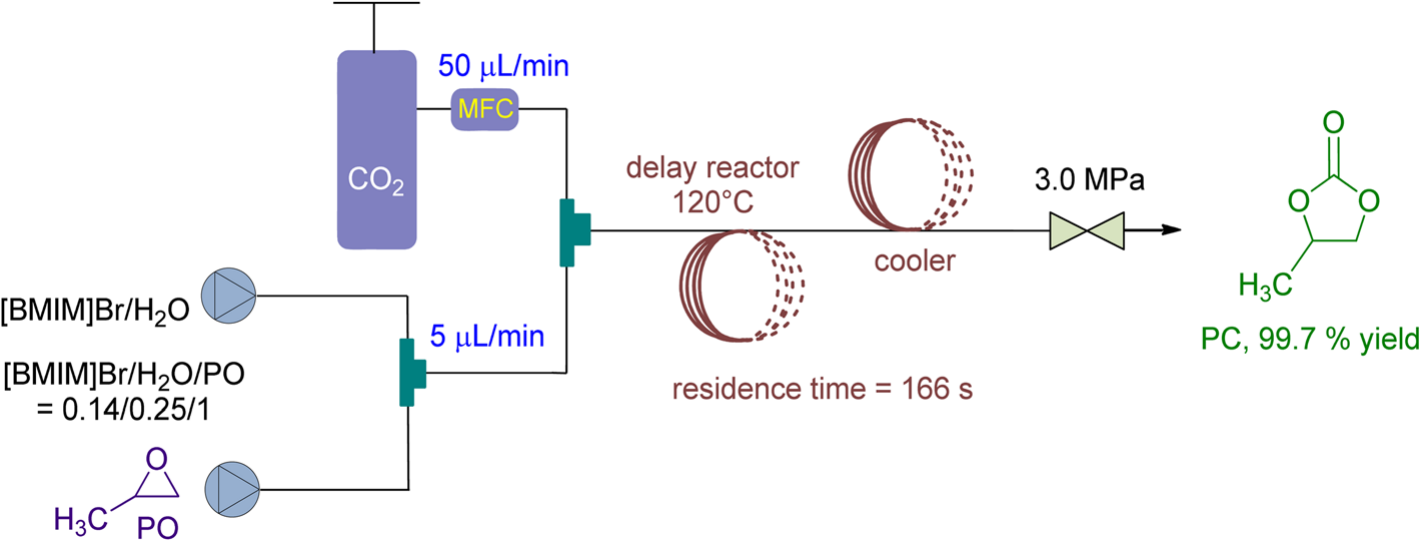
Propylene oxide and CO_2_ coupling are coupled by [BMIM]Br (reproduced from ref. 138 with permission from Elsevier copyright 2021).

In 2021, Wu *et al.*^138^ stated the use of a micro reaction system in a continuous flow setup for CO_2_ fixation, using 1-butyl-3-methylimidazolium bromide ([BMIM]Br) and H_2_O as catalyst and co-catalyst, respectively (Fig. 16). At 3 MPa and 140 °C, they achieved 90 to 99.8% conversion with a residence time of just 166 seconds. The molar ratio of [BMIM]Br/H_2_O/PO was set at 0.14/0.25/1, and the CO_2_/PO molar ratio was 1.4. Additionally, the recycling performance of [BMIM]Br catalyst was examined, yielding over 86% even after being recycled and repurposed 5 times without losing selectivity.

**Fig. 17:**
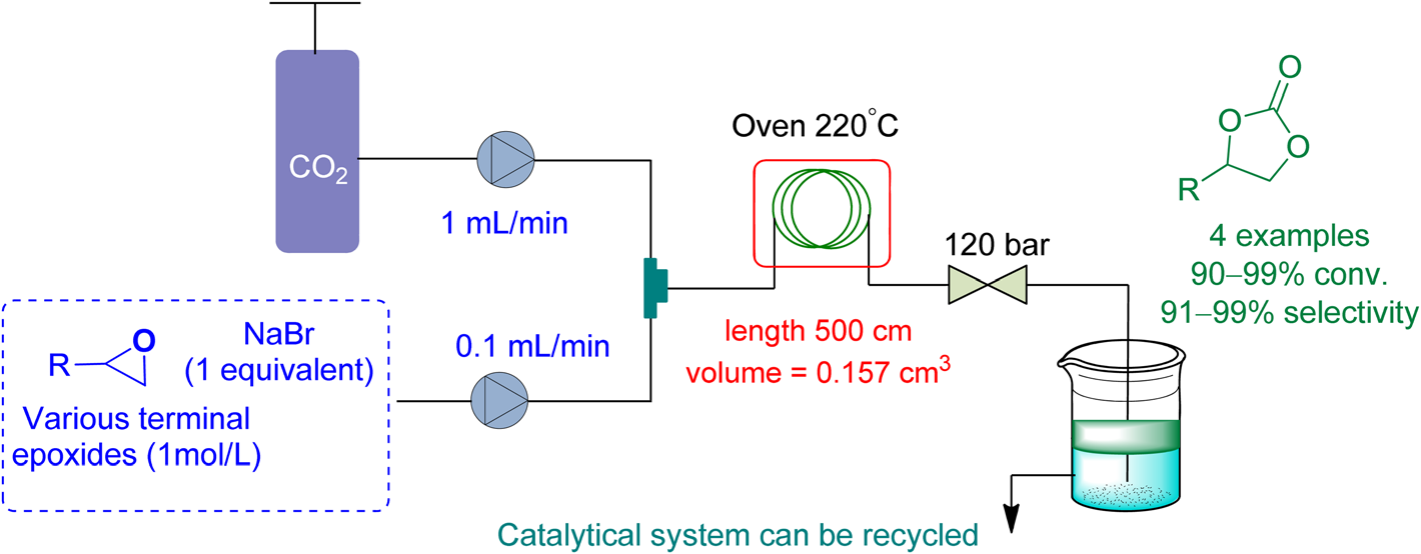
Propylene oxide and CO_2_ are coupled using diethylene glycol/NaBr system catalysis (reproduced from ref. 165 with permission from Wiley-VCH copyright 2021).

In 2021, Rigo *et al.*^165^ used a micro-fluidic reactor to perform cycloaddition to epoxides with a binary system NaBr/diethylene glycol (DEG) as a catalytic system, using DEG as a reaction medium (Fig. 17). The flow rate of epoxide and CO_2_ was kept at 0.1 mL min^−1^ and 1.0 mL min^−1^, respectively. At 120 bar and 220 °C, they obtained a conversion of 91 to 99% for a range of cyclic carbonates.

**Fig. 18:**
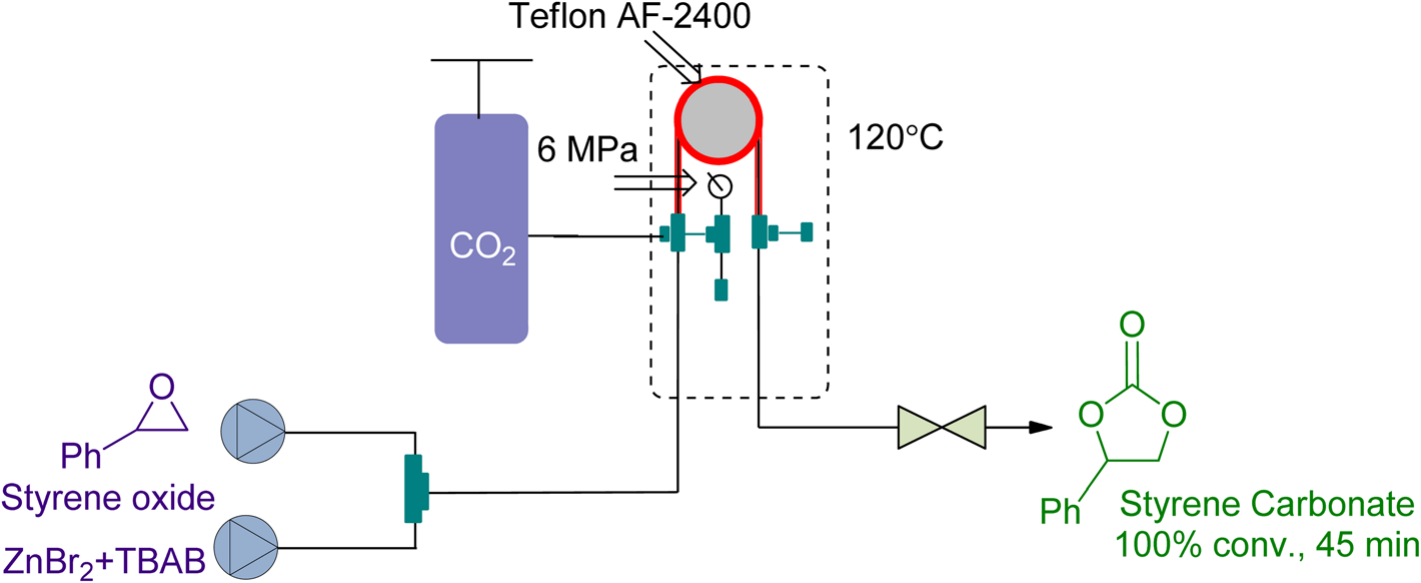
Styrene oxide and CO_2_ coupling using a synergistic system of ZnBr_2_/TBAB (reproduced from ref. 166 with permission from Elsevier, copyright 2018).

Tube-in-tube reactors are gaining attention as an intelligent process for gas–liquid transformations. In 2018, Rehman *et al.*^166^ studied the CO_2_ cycloaddition of styrene oxide (SO) using a ZnBr_2_/TBAB catalytic system in a gas–liquid continuous flow reactor of this type. They achieved 100% conversion at 6 bar and 120 °C in 45 min (Fig. 18). The kinetic study showed that it was a first-order reaction with respect to the reactants. The decrease of activation energy by 23 kJ mol^−1^ using ZnBr_2_ as a co-catalyst highlighted the importance of synergy.

**Fig. 19:**
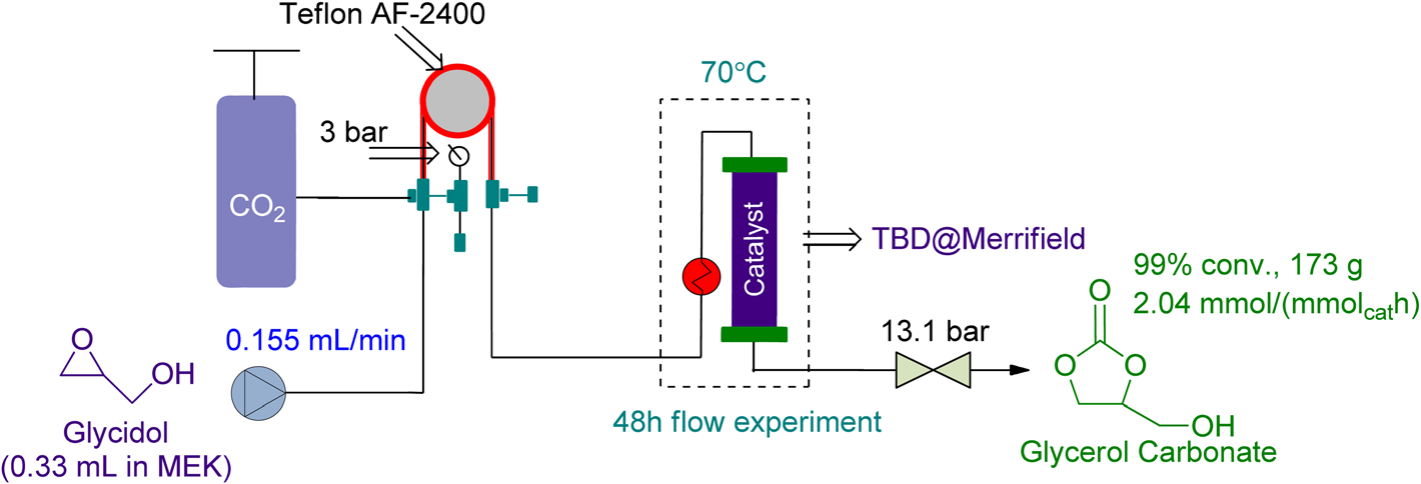
Coupling reaction in a tube-in-tube reactor using TBD@Merrifield as a catalyst (reproduced from ref. 113 with permission ACS from copyright 2021).

In 2021, Zanda *et al.*^113^ used a tube-in-tube reactor followed by a packed bed reactor in series for the preparation of GC from Gly. They used 1.59 g of heterogeneous TBD @ Merrifield as a catalyst and MEK as a solvent (Fig. 19). In 48 h, 99% conversion was attained while the reaction was being conducted at 70 °C and 3 bar of CO_2_ pressure.

### 3.3 Synthesis of limonene carbonate using a flow reactor

A novel continuous flow method for converting 1,2-LO and LDO to limonene cyclic carbonates was recently reported.^91^ Supercritical CO_2_ was the only reagent and solvent used in the experiment, and easily producible heterogeneous supported ionic liquid phase (SILP) catalysts were applied. ILs are excellent candidates for use as reaction media in catalytic processes due to the high solubility of supercritical CO_2_ in them. SILP materials have a high surface area that is beneficial for catalytic processes and are made up of a thin layer of ionic liquid on a supporting material, such as mesoporous silica. This enables the researchers to overcome mass transfer limitations caused by short diffusion lengths in the thin film.

**Fig. 20:**
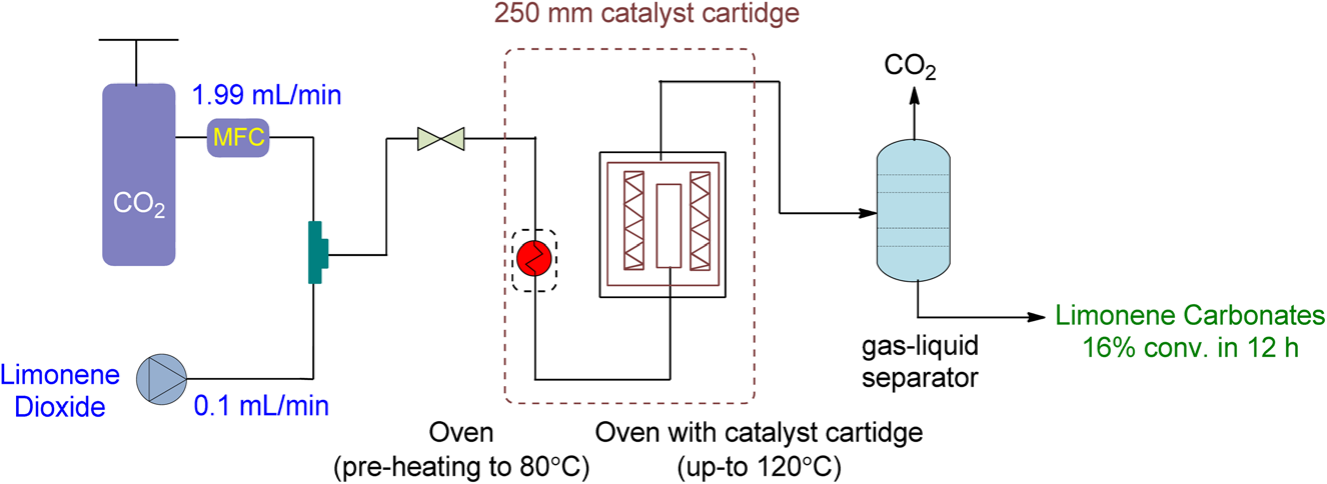
Supported Ionic liquid (SLIP) catalysed heterogeneous reaction of limonene oxide and CO_2_ (reproduced from ref. 91 with permission from ACS, copyright 2022).

**Fig. 21:**
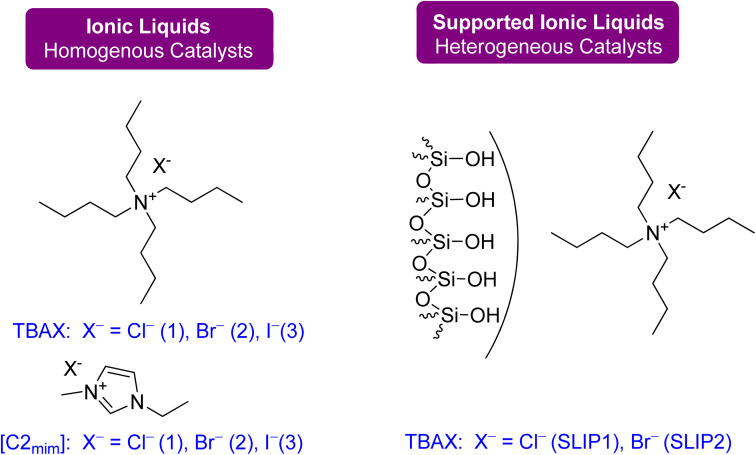
Catalysts: ammonium- and imidazolium-based ionic liquids were used as homogeneous catalysts, and SILPs were applied as heterogeneous catalysts for flow synthesis (reproduced from ref. 91 with permission from ACS, copyright 2022).

The setup for continuous flow reaction is shown in Fig. 20. Both scCO_2_ and substrate were mixed and sent to the preheater when a temperature greater than 80 °C was required, followed by a second heating unit to heat the catalyst cartridge to 150 °C. The catalyst input for two different cartridge lengths (150 and 250 mm) was 1.34 and 2.22 g of SILP material, respectively. Back-pressure regulators were used to enabling reactions to occur at different pressures. The final product was collected after passing through a gas–liquid separator, containing carbonates and unreacted starting material. Optimization was performed on the temperature, pressure, catalyst loading, and flow rates of CO_2_ and substrates. The reaction required a 2–3 h preliminary lead time under the low flow rate of 1,2-LO of 0.01 mL min^−1^. The SILP catalysts used were easily producible heterogeneous and best-performing supported ionic liquid phase (SILP) catalysts (Fig. 21).

Various factors were studied to optimize the continuous flow process for the conversion of 1,2-LO and LDO to limonene cyclic carbonates. Firstly, an increase in temperature from 80 to 120 °C increased yield. However, at 150 °C, both degradation and Hoffmann elimination of TBAC 1 to tributylamine caused a decline in yield over time. Secondly, to prevent the leaching of the catalyst from its supporting material during prolonged industrial use, the corresponding pressure of 6 to 20 MPa was studied at 120 °C. It was observed that 12% leaching occurred at 6 MPa, while no leaching was observed at 15 and 20 MPa. However, at 20 MPa, product formation declined. Thirdly, a catalyst cartridge of 250 mm (residence time of 125 s) was used instead of 150 mm (residence time of 75 s), and the increased input of SILP to 2.22 g resulted in a 60% yield increment. Fourthly, catalyst loading of 20 to 40 wt% was studied, and it was found that a 30 wt% loading instead of 20 wt% increased the yield, while a 40 wt% loading was unsuitable and overburdened the pressure. Lastly, the flow rates of scCO_2_ and substrate (LO-*cis*/*trans*) were studied. The flow rate of CO_2_ between 1.99 and 2.49 mL min^−1^ was found to be optimum, while a flow rate of 0.99 mL min^−1^ caused choking and a flow rate of 3.99 mL min^−1^ caused leaching. Similarly, a higher flow rate of (LO-*cis*/*trans*) 0.02 L min^−1^ instead of 0.01 mL min^−1^ resulted in leaching.

Using a heterogeneous catalyst optimized under specific conditions (48 h reaction time, 15 MPa pressure, 120 °C temperature, 2.22 g of SLIP 1 catalyst input, 0.01 mL min^−1^ of LO-*cis*/*trans* substrate flow rate, 1.99 mL min^−1^ of CO_2_ flow rate, and a 250 mm catalyst cartridge), a maximum yield of 22% and an overall yield of 16% were achieved. This resulted in a novel production flow rate of 0.12 g h^−1^, which had not been reported before. To further optimize the process, future experiments could focus on using only the more reactive *cis* isomer for the chemical fixation of CO_2_ under flow conditions.

**Fig. 22:**
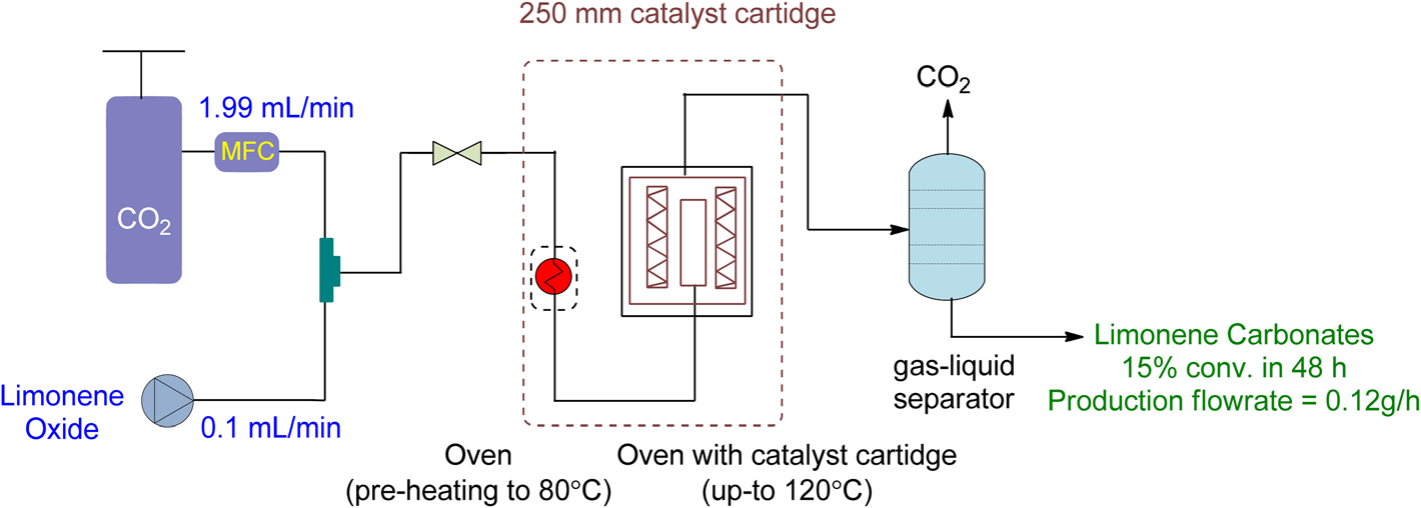
Supported Ionic liquid (SLIP) catalysed heterogeneous reaction of LDO and CO_2_ (reproduced from ref. 91 with permission from ACS, copyright 2022).

Diastereomeric mixtures of epoxy carbonate and bicarbonate can be formed by converting LDO, as shown in Fig. 22. Epoxy carbonate, being less sterically hindered, yields higher yields than bicarbonate. To optimize the reaction, a heterogeneous catalyst was used under specific conditions (12 h, 20 MPa, 120 °C, 2.22 g of SLIP 1, 0.01 mL min^−1^ of LO-*cis*/*trans*, 1.99 mL min^−1^ of CO_2_, 250 mm catalyst cartridge), resulting in a maximum yield of 27% for epoxy carbonate and 14% for bicarbonate, with an overall yield of 16%. Table 7 provides a summary of cyclic carbonates synthesis under flow conditions, showcasing the various types of reactors and their respective reaction conditions.

**Table 7:** Summary of cyclic carbonates synthesis under flow conditions

Substrate/epoxide	Epoxide flow rate	CO_2_ flow rate	Reactor type	Reaction/reactor conditions (*T*, *P*)	Catalyst req.	Conversion + time	Selectivity	Other reported	References
Propylene oxide (PO)	0.1 mL min^−1^	0.2 mL min^−1^	Fixed bed flow reactor	10 MPa, 90–160 °C	10 g SiO_2_– C_3_H_6_–P(*n*-Bu)_3_Br (8.5 mmol phosphorus)	80% in 1000 h	99.9%	WHSV = 0.5 per h, TON = 11 500, synergistic organic-inorganic catalyst	156
Epichlorohydrin	1.0 mL min^−1^	50.0 mL min^−1^	Packed bed reactor	1.4 MPa, 140 °C	Coconut shell-activated carbon (CSAC) tethered Bmim-COOH/Br	82% in 50 h	n.a	Liquid hourly space velocity (LHSV) = 6 per h	157
Propylene oxide (PO)	15 μL min^−1^		Fixed bed flow reactor	2 MPa, 130 °C	PSIL (IMD) imidazolium-based polymer-supported ionic liquids	41–45% in 130 h	n.a	GHSV 4000 per h	158
Styrene oxide (SO)	5 μL min^−1^	50 μL min^−1^	Continuous flow reactor	140 bar, 150 °C	Rose bengal supported ionic liquid-like phases (RB-SILLP)	53% in 10 days	99%	Productivity of 1.129	159
Propylene oxide (PO)	0.1 mL min^−1^	5.0 mL min^−1^	Packed bed reactor	2 MPa, 90 °C	DBU@SBA-15 (100 mg)	57.1% for 2 h continuous reaction/16.86% for 24 h	99%		160
Epichlorohydrin and 1,2 butylene oxide	0.01–0.04 mL min^−1^	15.0 mL min^−1^	Vertical fixed-bed reactor packed with the polymeric MMFR (continuous flow)	13 bar, 120 °C	Mesoporous melamine-formaldehyde resins MMFR (1.95 g, ≤100 μm) or MMFR 250 (1.5 g, ≤100 μm)	76–100% conversion in 7 to 13 days	99.9%	Weight hourly space velocity (WHSV) of 0.26–1.9 h^−1^	161
Propylene oxide (PO)	n.a	n.a	Microreactor	3.5 MPa, 180 °C	HETBAB	99.8% in 14 s	n.a		136
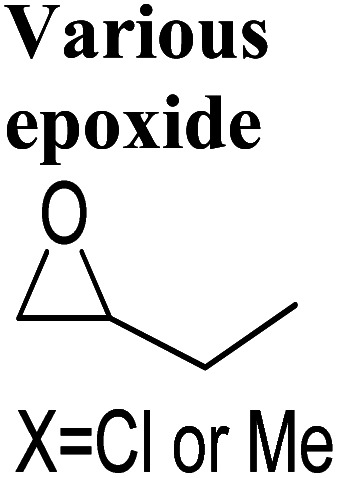	0.3–1.2 mL min^−1^	192.5–770 mL min^−1^	Microreactor followed by a delayed reactor	2 MPa and 150 °C	Binary (salen) AlCl/*n*Bu_4_NBr (TBAB) system	90–99% with a residence time of less than 100 s	99%	Residence time 100 s, TOF = 6800 to 14 700 h^−1^	164
Propylene oxide (PO)	n.a	n.a	Continuous-flow micro-reaction system	3 MPa, 140 °C	1-Butyl-3-methylimidazolium bromide ([BMIM]Br)/H_2_O	99.8%	n.a	Residence time 166 s, molar ratio of [BMIM]Br/H_2_O/PO to be 0.14/0.25/1, the molar ratio of CO_2_/PO to be 1.4	138
Terminal epoxide (R = *n*C_4_H_9_)	0.1 mL min^−1^	1 mL min^−1^	Microfluidic reactor	120 bar and 220 °C	NaBr/diethylene glycol (DEG)	91 to 99%	92 to 99%	Reaction medium (DEG)	165
Styrene oxide	n.a	n.a	“Tube-in-tube” gas–liquid continuous flow reactor	6 bar and 120 °C	Tetrabutylammonium bromide (TBAB)/ZnBr_2_ homogenous system	100% in 45 min	n.a	Activation energy decrement 55–32 kJ mol^−1^ = 23 kJ mol^−1^	166
Glycidol (Gly)	0.155 mL min^−1^	n.a	“Tube-in-tube” reactor followed by a packed bed reactor	3 (bar CO_2_ local pressure)	TBD@Merrifield (catalyst loading of 1.59 g/1.48 mmol)	99% in 48 h	n.a	MEK solvent	113
LO-(*cis*/*trans*)	0.1 mL min^−1^	1.99 mL min^−1^	Continuous flow reaction	15 MPa, 120 °C	Supported ionic liquid SLIP 1 (2.22 g) (Fig. 21)	22% (max) and 15% (overall) in 48 h	n.a	250 mm catalyst cartridge, production flow rate of 0.12 g h^−1^	91
Limonene dioxide (LDO)	0.1 mL min^−1^	1.99 mL min^−1^	Continuous flow reaction	20 MPa, 120 °C	Supported ionic liquid SLIP 1 (2.22 g) (Fig. 21)	27% (epoxy carbonate) 14% (bis-carbonate), and 16% (overall) in 12 h	n.a	250 mm catalyst cartridge	91

## 4. Summary

This review summarizes the latest developments in using CO_2_ to produce cyclic carbonates from bio-based precursors. Future research should focus on proposing catalytical systems that can reduce the temperature and pressure requirements for CO_2_ and epoxide reactions. The selection of product routes based on CCU is complex, and economic and environmental assessments of each route are necessary. Novel designs for integrating Capture and Utilization at the same station are anticipated, and research should aim to improve financial and energy penalties. Ethanol, methanol, and syngas production have high potential, but research and development efforts are needed to make them feasible. Processes that require hydrogen will need a sustainable or green method of hydrogen production for commercial viability. Process Intensification tools such as intensified equipment and processes can improve mass and heat-transfer rates. Over 50% of processes are laboratory-based, so industrialization efforts are necessary. Recent advances in flow chemistry have enabled the use of CO_2_ as a C1 synthon and packed-bed and microreactors have shown promise for coupling CO_2_ and epoxides. Further research should focus on developing robust catalytic systems, novel reactor designs, and efficient synthesis techniques. With the growing number of publications in this field, a bright future is forecasted, and this review provides a starting point for further advancements.

## Conflicts of interest

There are no conflicts to declare.

## Supplementary Material
